# Synthesis and Properties of Star-Shaped Hydroxyl-Terminated Polybutadiene via RAFT Polymerization: A Model for a Binder Prepolymer

**DOI:** 10.1021/acsomega.5c06683

**Published:** 2025-11-03

**Authors:** Wesley S. Farrell, Edward Gravois, Nicholas C. Molineaux, Anthony M. Clay

**Affiliations:** † Chemistry Department, 32722 United States Naval Academy, 572M Holloway Rd, Annapolis, Maryland 21402, United States; ‡ DEVCOM, FCDD-RLW-M, Polymers Branch, Aberdeen Proving Ground, 6300 Roadman Road, Aberdeen, Maryland 21005, United States

## Abstract

Fundamental knowledge on how polymer architecture affects curing and material properties of solid rocket motors (SRMs) using hydroxyl-terminated poly­(butadiene) (HTPB) as a prepolymer has historically been based on studies employing impure samples. Herein, we present the synthesis of highly controlled HTPB via reversible addition–fragmentation chain-transfer (RAFT) polymerization and explore how the hydroxyl group content affects viscosity and pot-life. We further examine the kinetics of curing in order to gain a mechanistic insight. The synthesis of these polymers involved the design and preparation of chain-transfer agents, which allowed for star-shaped polymers via the Z-group approach. We demonstrate that increasing the number of hydroxyl groups serves to decrease the pot-life, despite the fact that network forming reactions (e.g., urethane formation) counterintuitively proceed more slowly, providing insight into the mobility of reactive chain ends during curing reactions relevant to SRM loading. Further, the architecturally pure materials presented here all have longer pot-lives than the commercially obtained HTPB, highlighting the benefit of using more controlled polymers for energetics applications. This represents the first examination of these processes using architecturally pure HTPB, a rare example of homopolymerization of butadiene using RAFT polymerization and a facile approach to more complex structures of poly­(butadiene) than have been reported previously.

## Graphical abstract

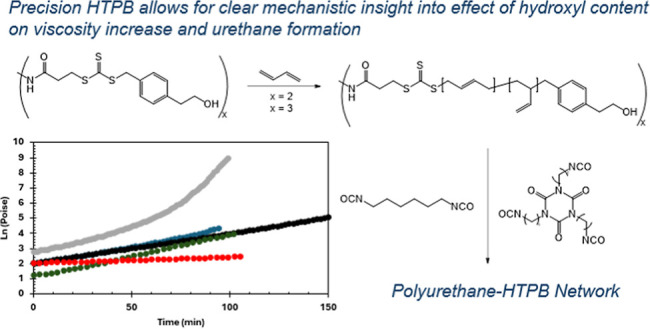

## Introduction

Solid rocket motors (SRMs) find use in propulsion for space exploration, jet-assisted takeoff, and military applications; are preferred above liquid fuel due to their increased safety and reliability; and do not require an external oxidizer. The motor is composed of metal, an oxidizer, and a binder. The binder is a polymer network that, upon forming via cross-linking, provides shape and stability and prevents void formation. The binder may be generated from an energetic prepolymer or one that is a pure hydrocarbon, which is under-oxidized and acts as a fuel.^1^ Desirable features for a binder include uniform distribution of solid particles, which can be improved by lengthening pot-life (i.e., the time required for viscosity to reach a point at which further mixing is not possible), favorable mechanical properties, high solids loading, and easy processability.^1,2^ Low viscosity of the prepolymer is a key feature for achieving these goals.

Much research has been done on the synthesis of energetic prepolymers for use in binders given that they contain explosophors (i.e., energetic functional groups) which generate significant amounts of energy and gas compared to their inert counterparts.^1,3,4^ For example, hydroxyl-terminated poly­(butadiene) (HTPB) may be nitrated using dinitrogen pentoxide,^5^ which generates prepolymers of sufficiently low viscosity to allow for processing.^6^ A recent report describes a creative method for postpolymerization modification of HTPB using reversible addition–fragmentation chain-transfer (RAFT) polymerization and macromolecular design by interchange of xanthates (MADIX), in which xanthates are added to pendant vinyl groups, or cross-linking can be induced.^7^ Explosophors were not added, and doing so is beyond the scope of this manuscript but may be possible and should be explored. Glycidyl azide polymer is prepared by the reaction of low molar mass poly­(epichlorohydrin) with sodium azide.^8^ Many other energetic polymers have been reported, and the topic has been reviewed.^1,3,4^

HTPB, an inert binder, is one of the most common prepolymers employed in SRMs.^1^ It is cured with aliphatic di/triisocyanates to make polyurethane networks. While it has been employed for over 60 years, research into HTPB continues unabated, in particular with regard to its curing. For instance, it has been shown recently that bioderived rosin esters may be incorporated during curing to give significant modifications to the end properties, such as large increases in elongation at break,^9,10^ or that other changes in mechanical properties can be observed based on curing solvent or HTPB functionalization.^11^ HTPB is prepared by free radical polymerization of butadiene in an alcoholic solvent (sometimes with water) using hydrogen peroxide as an initiator. In this type of polymerization, there is little control over the polymer structure. Specifically, the molar mass distribution (MMD) is broad and often bimodal, and the amount of hydroxyl groups in any given HTPB macromolecule is not uniform across these molar mass populations as a result of 1,2-additions during the uncontrolled polymerization.^12,13^ These uncertainties are the cause for myriad problems. The most obvious is that the lack of control leads to variations in each batch of HTPB prepared, making the resulting filled binder system inconsistent from one SRM to another. A bimodal MMD is undesirable, as small populations of high or low molar mass materials can impact bulk properties. More obviously, one can imagine that an increase in the number of hydroxyl groups would lead to faster network formation, thereby shortening the pot-life. Indeed, it has been demonstrated that an increase in both molar mass and hydroxyl content leads to faster viscosity build-up during curing (although these variables were not isolated, and the materials were enriched in chains with higher hydroxyl content, but in no way pure).^2^ In another analysis, it was shown that decreasing molar mass and hydroxyl content allows for a higher elongation at break, another favorable property.^12^ In both of these works, however, the amount of hydroxyl groups is determined by ^1^H and ^13^C nuclear magnetic resonance (NMR) spectroscopy and size-exclusion chromatography (SEC) and represents average values. Assumptions are made that no single macromolecule contains more than 3 hydroxyl groups, but in reality, it is known that some HTPB molecules will contain many more.^13^ Given that small amounts of structural impurity or high/low molar mass populations can have outsized impacts on bulk properties, these average values are useful but by no means conclusive.

Motivated by this gap in knowledge, we were inspired to prepare HTPB in a more controlled manner, in terms of both MMD and hydroxyl group content. An attractive way to prepare such polymers is through the synthesis of star-shaped HTPB. Unfunctionalized poly­(butadiene) stars and H-shaped polymers have been prepared extensively over the years using anionic polymerization,^14–18^ with as many as 270 arms reported. As expected, viscosity decreases and entanglement molar mass increases as the number of arms increases.^19–21^ Inclusion of hydroxyl end-groups was not reported. Atom-transfer radical polymerization of butadiene has long been an elusive goal and has recently been reported for both linear and star-shaped poly­(butadiene),^22^ but again the inclusion of hydroxyl end-groups was not reported and would require postpolymerization modifications, which may not yield 100% functionalization. We desired a method of controlled polymerization that lacked postpolymerization modification for the preparation of star-shaped HTPB, in order to streamline the synthesis and eliminate the possibility of incomplete hydroxyl group installation.

RAFT polymerization stood out as an attractive method, particularly given its control and mild conditions.^23^ In this polymerization (Scheme 1A), a radical initiator begins the polymerization of the butadiene monomer. The growing polymer is then transferred to a chain-transfer agent (CTA), producing a new radical, R^•^. The R^•^ group then initiates further polymerization and transfers back to the CTA, establishing an equilibrium between active and dormant species. Other types of initiation have been reported more recently, such as aerobic mechanical-induced initiation,^24^ photoinduced initiation electron transfer,^25^ or photoinerferter mechanisms.^26^ RAFT is attractive for functionalized star-shaped polymers because the CTA can be designed such that the Z group (which is nonlabile) is attached to a core molecule, while the R group contains the desired hydroxyl functionality. This is called the “Z-group approach” and is favored over the “R-group approach”, in which the R-group is tethered to the core, for small stars, as it prevents star–star coupling and diffusion of growing radicals to the core is not inhibited at low molar mass (Scheme 1B).^27^ RAFT has been sparingly used in the polymerization of butadiene previously,^28^ including one instance of unfunctionalized star polymers and copolymers.^29^ In the present work, we prepared CTAs bearing 2 and 3 arms in which the R-group was functionalized with an aliphatic alcohol and employed them in the synthesis of HTPB. While a 2-arm star represents a linear polymer, we include this as a key reference given the large amount of linear HTPB used in SRMs and refer to it as a star for consistency’s sake while recognizing the shortcoming of this nomenclature. We studied materials properties relevant to binder performance, namely, viscosity and pot-life, and kinetics for curing with different isocyanate cross-linkers. We find that the 3-arm star is less viscous before curing compared to the 2-arm star but that its viscosity builds much more quickly than its 2-arm counterpart due to the additional hydroxyl group capable of undergoing urethane linkage formation. Additionally, the rate of urethane formation is lower for the 3-arm star, likely due to the fact that after the first 2 hydroxyls react, the ability for the third to react is hampered due to slower diffusion in the viscous network. This is the first demonstrative example of how hydroxyl group content affects these key properties and should be used to guide HTPB synthesis moving forward.

**Scheme 1:**
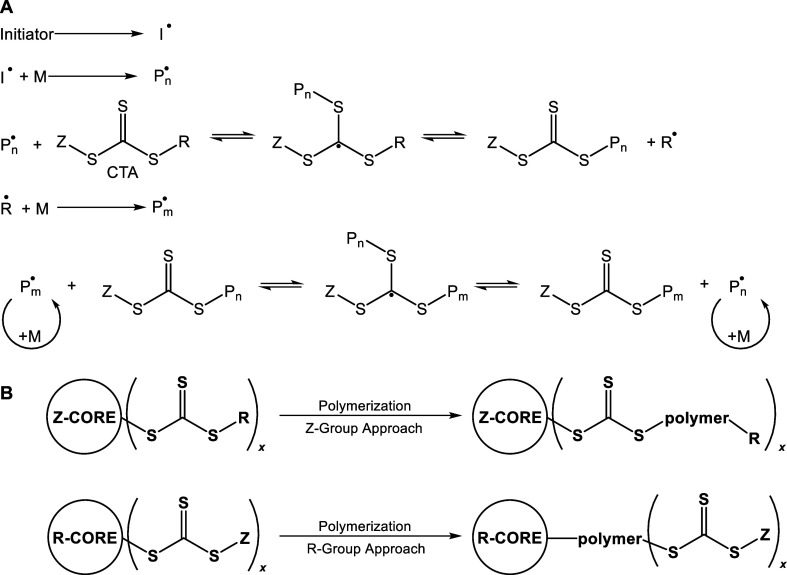
(A) Mechanism of RAFT Polymerization (*P*_n/m_ = Polymeryl and M = Monomer), (B) Z-Group and R-Group Approaches to the Synthesis of Star Polymers via RAFT

## Results and Discussion

### Synthesis of Chain-Transfer Agents

To begin, appropriate CTAs were needed to be synthesized. Stenzel and co-workers have demonstrated the use of the benzyl-substituted trithiocarbonate 3-benzylsulfanylthiocarbonylsulfanylpropionic acid, accessible in one step from commercially available materials, to mediate RAFT of acrylates, acrylamides, and styrene to yield both linear polymers^30^ and, upon modification of the CTA, star polymers and dendrimers.^31,32^ We reasoned that this would be an attractive scaffold on which to prepare a CTA for the desired HTPB star polymers given the ease of coupling of the carboxylic acid to a core and modification of the benzyl group. To this end, as shown in Scheme 2, we prepared alcohol **2** from the commercially available carboxylic acid **1** according to a modified literature procedure,^33^ quenching the reduction with 1 M H_2_SO_4_ rather than water. The alcohol was protected to form compound **3**, followed by the introduction of the trithiocarbonate to form compound **4**. In our hands, use of aqueous potassium hydroxide to deprotonate the thiol was unsuccessful, and consistently gave 0% yields.^30^ However, mild heat using K_3_PO_4_ in acetone produced **4** in high yield.^34^ Coupling to an amine core yielded **5a**–**b** in moderate yield using dicyclohexylcarbodiimide (DCC) with catalytic dimethylaminopyridine (DMAP), followed by deprotection with 1 M HCl in THF to yield **6a**–**b** in moderate yields following purification. NMR analysis confirmed that the desired structures were obtained, and specifically, ^1^H NMR clearly indicated that the desired hydroxyl groups were present in each CTA.^35^ Deprotection with tetrabutylammonium fluoride yielded a complex mixture of unidentified products.

**Scheme 2:**
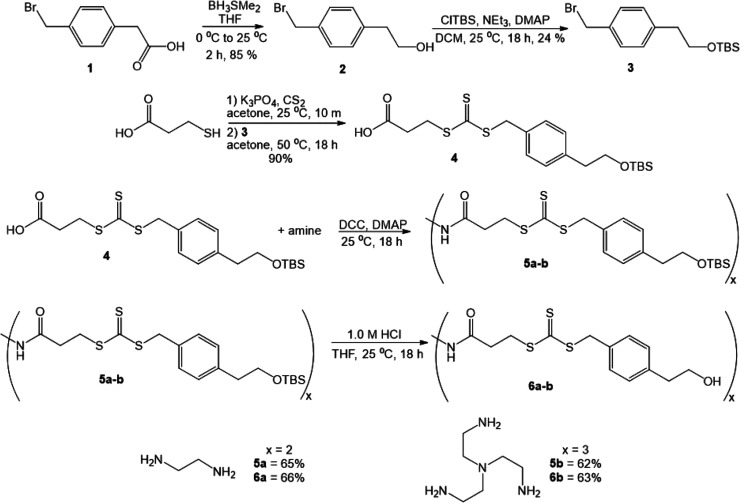
Synthesis of CTAs **6a**–**b**

### Synthesis of Polymers

Polymerizations were performed at 95 °C for 3 days by adding gaseous butadiene to toluene/DMSO mixtures of **6a**–**b** with dicumyl peroxide (DCP) as an initiator to yield 2-arm and 3-arm star polymers **7a** and **7b**, respectively (Figure 1A). Initially, 0.5 equiv. DCP per trithiocarbonate was employed, a ratio chosen based on the kinetics studies of polymerization of butadiene with a similar trithiocarbonate CTA shown to shorten the reaction times.^36^ Toluene has been used as a solvent in similar polymerizations;^36^ however, the CTAs used here are insoluble in everything but DMSO after purification. A target molar mass for the polymer of ∼3000 g/mol was desired. However, given that butadiene addition is judged by the pressure of the headspace and the addition was done to a degassed solution, this was difficult to achieve based on prescribed ratios and stoichiometry. We were able to determine appropriate pressures and [M]_0_/[CTA]_0_ ratios through trial-and-error and ensuring quick addition of monomer and sealing of the reactor.

**Figure 1:**
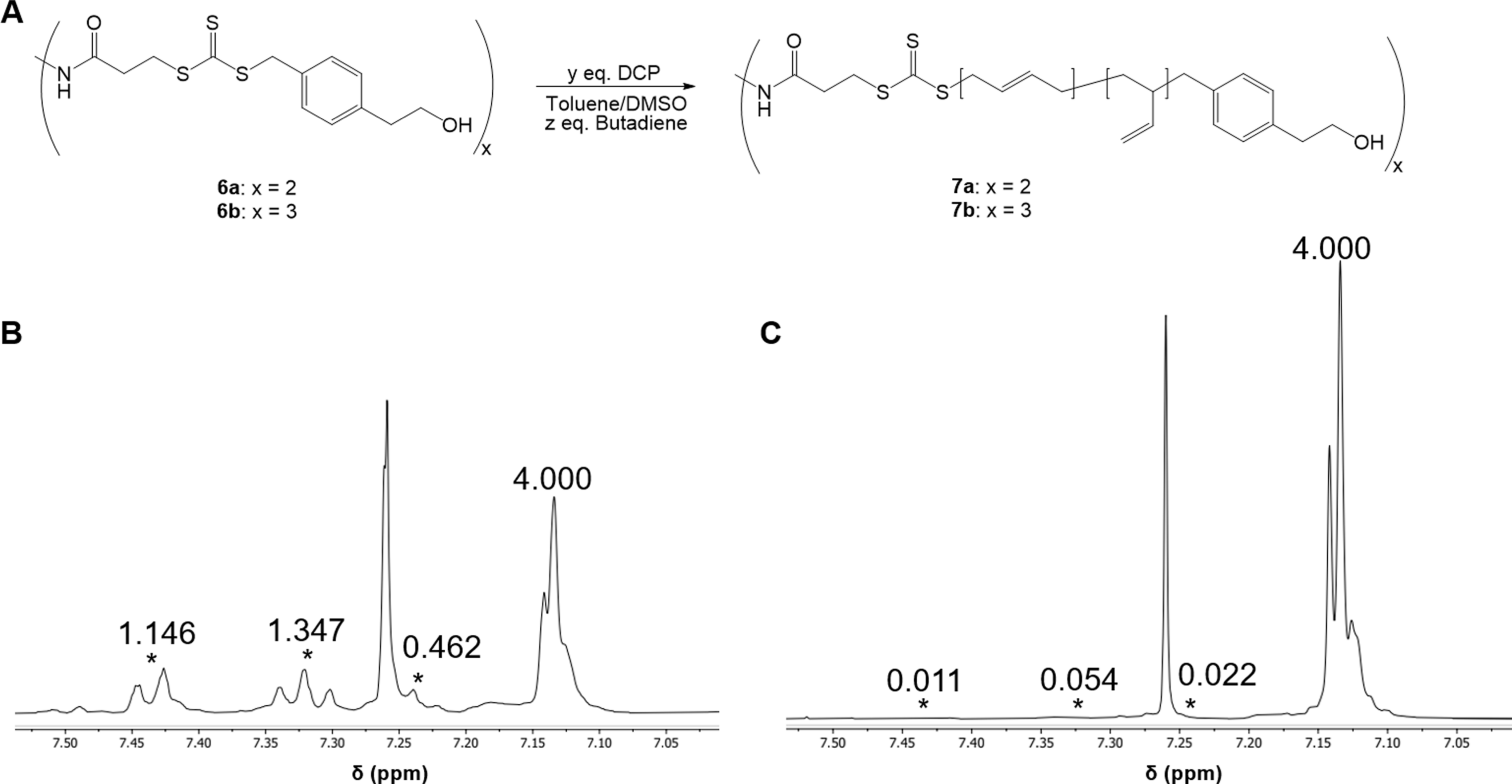
(A) Polymerization scheme for **7a**–**b** (both 1,4-additions and 1,2-additions are shown to better depict the structure of the resulting polymer). (B) Downfield region of the ^1^H (400 MHz, chloroform-*d*, 25 °C) NMR of **7a** made with 0.5 equiv. DCP, revealing extensive end-group impurity (DCP labeled with *, and integrations shown relative to the 4H signal of the aryl ring derived from the CTA). (C) Downfield region of the ^1^H (400 MHz, chloroform-*d*, 25 °C) NMR of **7a** made with 0.05 equiv. DCP, revealing minimal end-group impurity (DCP labeled with *, and integrations shown relative to the 4H signal of the aryl ring derived from the CTA).

^1^H NMR analysis of the resulting polymers revealed extensive incorporation of DCP-derived end-groups as a result of the large amount of DCP added (Figure 1B).^35^ To prevent this lack of end-group fidelity, we decided to lower the amount of DCP to 0.05 equiv per trithiocarbonate unit. To explore optimal conditions, we ran a series of butadiene polymerizations using the established CTA trithiocarbonate 3-benzylsulfanylthiocarbonylsulfanylpropionic acid given that it is structurally analogous to the star CTAs employed here but available in high yield in one step from inexpensive commercially available reagents.^30^ We found that in order to ensure maximum yield while minimizing DCP-derived end-groups, it was best to also increase the reaction temperature to 120 °C and the reaction time to 6 days. Temperatures above 120 °C resulted in lower yields, and longer times beyond 6 days did not increase yield (results and spectra are provided in the Supporting Information).^35^ In a subsequent polymerization using CTA **6a**, we found negligible incorporation of DCP-derived end-groups in **7a** (Figure 1C).

With this knowledge, we proceeded with the synthesis of the desired HTPB star polymers using CTAs **6a**–**b**, with a target [M]_0_/[CTA]_0_ of 77:1. Given the limitation in the size of the reactor, the polymerizations were repeated several times to generate enough material for analyses upon combination. All yield and molar mass data for repeated polymerizations is summarized in Table 1. Generally speaking, molar masses were found to be in the desired range for the analysis. Dispersity values were lower for those generated by 2-arm CTA **6a** than 3-arm **6b**, and the molar masses were more consistent across trials. Molar masses for the 3-arm star polymers **7b** were generally found to be lower than expected, especially compared to their linear counterparts **7a**. The reason for this disparity is likely the fact that **7b** possesses a smaller hydrodynamic radius in solution compared to linear polymers of the same molar mass, given its nature as a star polymer. Thus, it will elute later from the SEC column. When compared against linear standards using RI detection, this will present as a lower molar mass than is truly present.

**Table 1:** Polymerization Data Using Initiators **6a**–**b** ^a^

CTA	theor. *M*_n_ (g/mol)^b^	*M*_n_ (g/mol)^c^	*D̵* ^c^	yield (g)	% 1,2-addition^d^
**6a**	4799	3599	1.329	0.4060	19.4
**6a**	4803	4256	1.375	0.4125	19.2
**6a**	4832	4686	1.290	0.3825	19.2
**6a**	4824	3947	1.311	0.3427	19.2
**6b**	5218	4022	1.613	0.3008	19.2
**6b**	5182	2731	1.549	0.4916	19.4
**6b**	5179	3709	1.466	0.6151	19.2
**6b**	5205	2409	1.327	0.3633	19.3

a Butadiene/CTA molar ratio of Y:1 in toluene/DMSO at 120 °C for 6 days, with target of *Y* = 77.

b Calculated as (*M*_butadiene_ × *Y*) + *M*_CTA_.

c Determined by SEC with THF as an eluent at 25 °C, calibrated against narrow dispersity polystyrene standards.

d Determined by ^1^H NMR analysis of the relative integration of alkene signals of 1,4-poly­(butadiene) and 1,2-poly­(butadiene).

Structural analysis of the polymers was also carried out. Infrared (IR) spectroscopy clearly shows the presence of the terminal hydroxyl group (ν_OH_ = 3295 cm^–1^ for **7a** and ν_OH_ = 3283 cm^–1^ for **7b**), the carbonyl group of the core (ν_C=O_ = 1650 cm^–1^ for **7a** and ν_C=O_ = 1641 cm^–1^ for **7b**), and trithiocarbonate of the core (ν_C=S_ = 1062 cm^–1^ for **7a** and ν_C=S_ = 1059 cm^–1^ for **7b**).^35^ ^1^H and ^13^C NMR showed clearly the presence of both 1,4- and 1,2-additions (see Figure 2B for ^1^H NMR of **7a** and the Supporting Information for all other NMR spectra). For **7a**, the N–H broad resonance is visible; however, this signal is not seen in **7b**. For both polymers, no DCP-derived chain ends are observed, indicating fidelity of the hydroxyl end-group. For **7a**, the ^13^C NMR spectrum (Figure 1A) shows both the amide and trithiocarbonate signals of the CTA. Relative integration of the aryl protons of the chain end to the aliphatic poly­(butadiene) protons provided molar masses near those obtained by SEC. It should be noted that while values derived from ^1^H NMR provide further insight into the polymer structure, namely, the inclusion of the –OH-derived end-groups, they are inherently challenging to compare to molar masses derived from SEC, as the latter are determined by calibration against linear poly­(styrene) standards, while our polymers are poly­(butadiene) and, in the case of **7b**, not linear. For the ^1^H NMR of **7a** shown in Figure 1, the molar mass determined by SEC was 4370 g/mol, while by NMR, it was 3817 g/mol and for **7b**, a similar analysis gave a molar mass of 3658 g/mol by SEC, and 4434 g/mol by NMR.^37^ The fact that the molar mass determined by ^1^H NMR for **7b** is higher than that determined by SEC is again not surprising given that a 3-arm star polymer will have a smaller hydrodynamic radius compared to a linear version of the same polymer, and elute later, giving the appearance of a lower molar mass. Taken as a whole, the lack of DCP-derived signals in the NMR spectra, the close molar masses determined by NMR relative to SEC, the presence of amide and trithiocarbonate resonances by ^13^C NMR, and the IR spectra all indicate the production of controlled HTPB with either precisely 2 or 3 hydroxyl groups per macromolecule.

**Figure 2:**
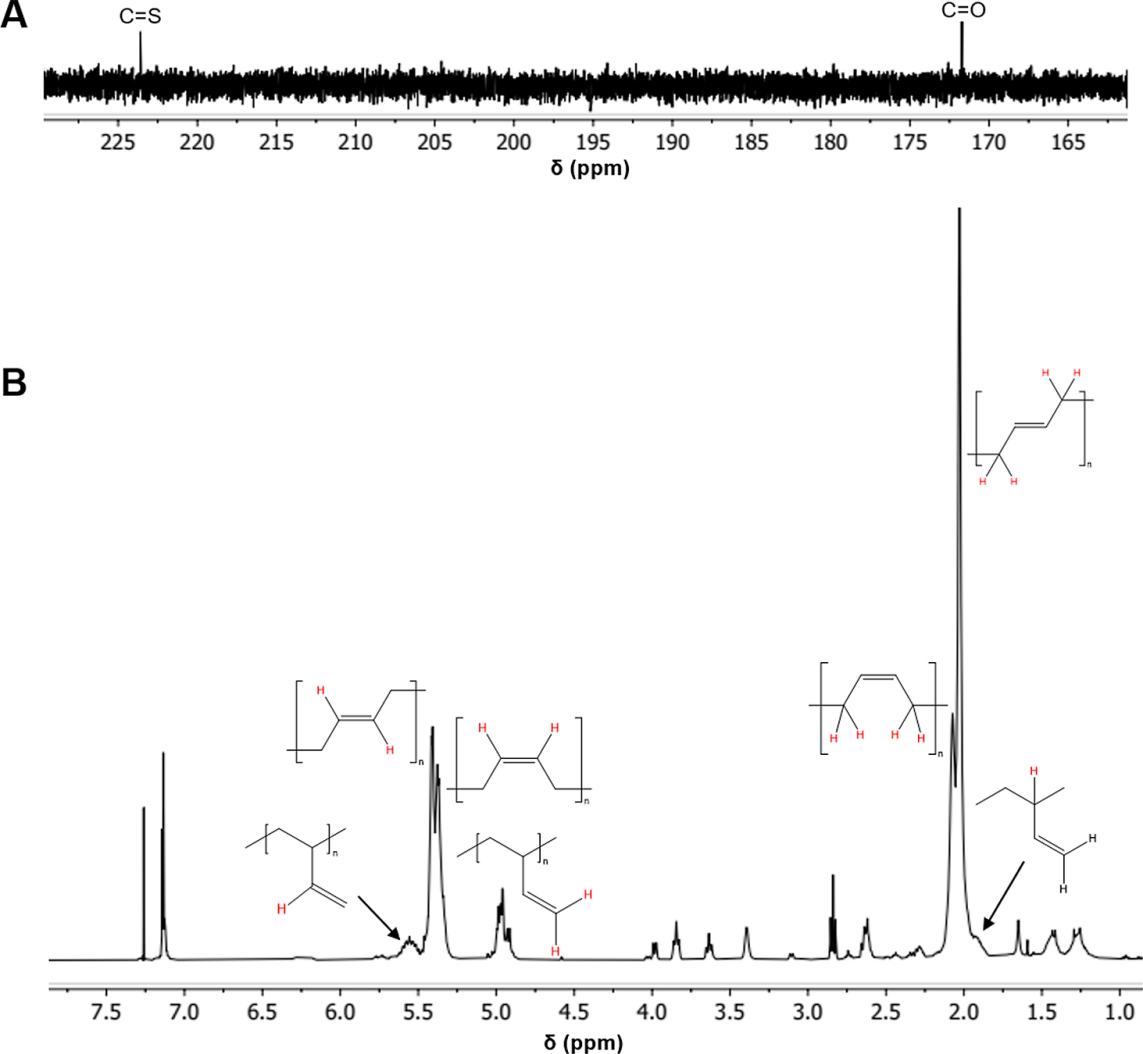
(A) Carbonyl region of ^13^C (100 MHz, chloroform-*d*, 25 °C) NMR of **7a**. (B) ^1^H (400 MHz, chloroform-*d*, 25 °C) NMR of **7a** (Note: Only signals relevant to the polymer are labeled, while those attributable to the CTA between 2.5 and 4.0 ppm and in the aromatic region are left unlabeled for clarity’s sake. Integral values are omitted for clarity. See the Supporting Information for the integrated spectrum).^35^

Lastly, in order to demonstrate that the polymers **7a**–**b** produced truly are stars, both were subjected to reaction with excess azobis­(isobutyronitrile) (AIBN) in toluene at 80 °C. In the case that the polymers were not as presented, one would expect no change in molar mass. However, if a decrease in molar mass is observed as evidenced by an increase in retention time in SEC, it would indicate that stars were present. In both cases, such an increase in retention time was observed by SEC.^35^

### Comparing Viscosities

Table 2 displays the viscosities of the individual hydroxyl compounds and isocyanates. The measured and reported viscosities of HTPB are similar. The viscosity of the trifunctional isocyanate Desmodur N3300A (Chart 1) is quite higher than that reported in the technical data sheet from the manufacturer, likely indicating that some aging occurred. The purchased HTPB has the highest viscosity at room temperature, while **7a** has a higher viscosity than **7b**, 4890 and 2595 mPa s, respectively, which is unsurprising as viscosity decreases with increasing number of arms for star polymers. As expected, increasing temperature decreased viscosity; Table 2 displays viscosities of the mixtures at 25 and 60 °C. The mixture **7b-**hexamethylenediisocyanate (HDI, a difunctional isocyanate, Chart 1) features the lowest mixture viscosity at 339 mPa s. HTPB-Desmodur N3300A features the highest mixture viscosity at 1616 mPa s. Interestingly, both **7a**-Desmodur N3300A and **7b**-Desmodur N3300A feature similar viscosities at 776.7 and 756.8 mPa s, respectively. This is also similar to the HTPB-HDI viscosity of 775 mPa s. All formulations feature a sufficiently low working viscosity below 1650 mPa s.

**Table 2:** Viscosities of Polymers, Isocyanates, and Mixtures

formulation	viscosity at 25 °C (mPa s)^a^	viscosity at 60 °C (mPa s)^a^	reported viscosity (mPa s)^b^
HTPB	5781 ± 3	1242 ± 4	5000 (30 °C)
			8000 (23 °C)
Desmodur N3300A	3800 ± 410	480 ± 40	1750–2250 (25 °C)
**7b**	2595	552.96	
**7a**	4890 ± 480	900 ± 50	
HDI			3
HTPB-HDI^a^		775.5	
**7b**-HDI^a^		339.3	
HTPB-Desmodur N3300A^a^		1616	
**7a**-Desmodur N3300A^a^		776.7	
**7b**-Desmodur N3300A^a^		756.8	

a Data taken from *t*_0_ of viscosity build kinetic plot.

b Obtained from technical data sheets from manufacturers.

**Chart 1:**
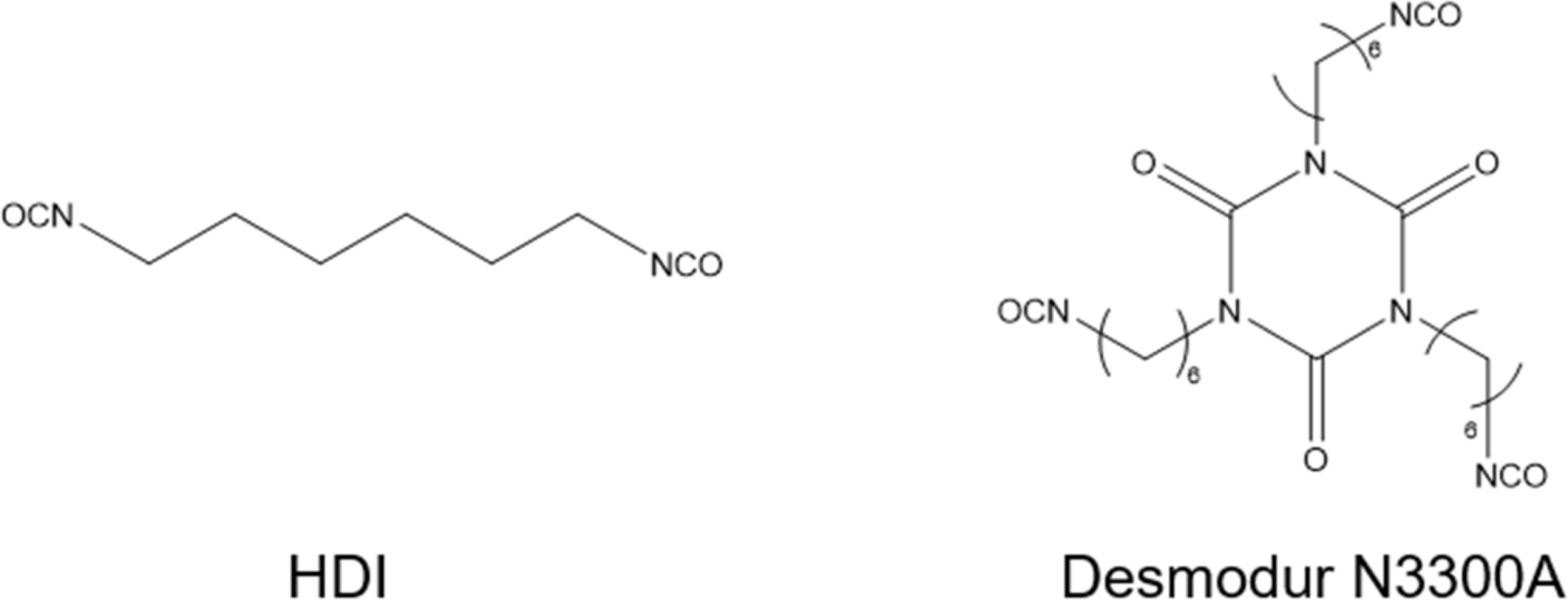
Isocyanate-Based Curing Agents

### Curing Experiments

In curing experiments, we employed both the difunctional isocyanate HDI and trifunctional Desmodur N3300A. Gelation experiments were performed to determine the time scale for kinetics experiments. Initial curing experiments were performed at 60 °C with commercially available HTPB to serve as a baseline measurement to compare synthesized derivatives. Gelation occurred as fast as 2 h and 8 min in the case of HTPB-Desmodur N3300A and required as long as 6 h and 10 min for HTPB-HDI. Figure 3 displays the rheological gel point of HTPB-HDI at 60 °C. These measurements gave a baseline working window for the following kinetic experiments.

**Figure 3:**
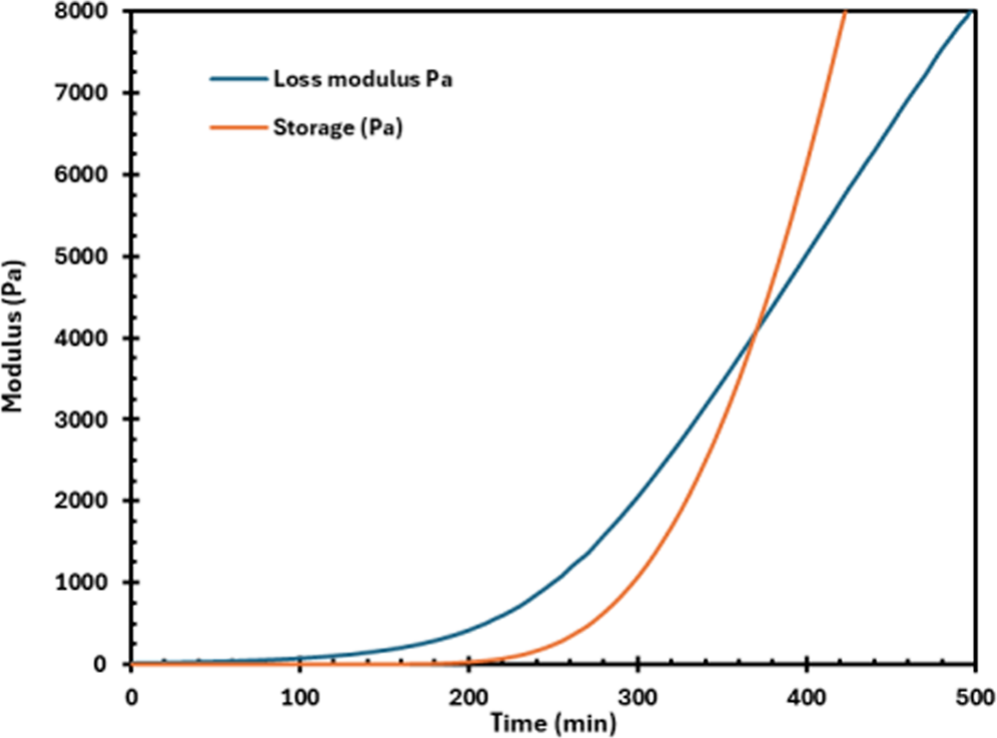
Rheological gel point determination of HTPB-HDI at 60 °C.

Both rheological and Fourier transform infrared (FTIR) spectroscopy kinetic experiments were conducted. Beginning with the rheological experiments, under steady flow shear rate at 60 °C, the viscosities of HTPB and the isocyanates were measured. Not surprisingly, trifunctional Desmodur N3300A led to faster viscosity increase as it provides more opportunities for cross-linking than the difunctional HDI. Across multiple trials for each, the average rate constants for viscosity increase for HTPB with Desmodur N3300A and HDI were 0.0335 min^–1^ and 0.221 min^–1^, respectively (Figure 4).

**Figure 4:**
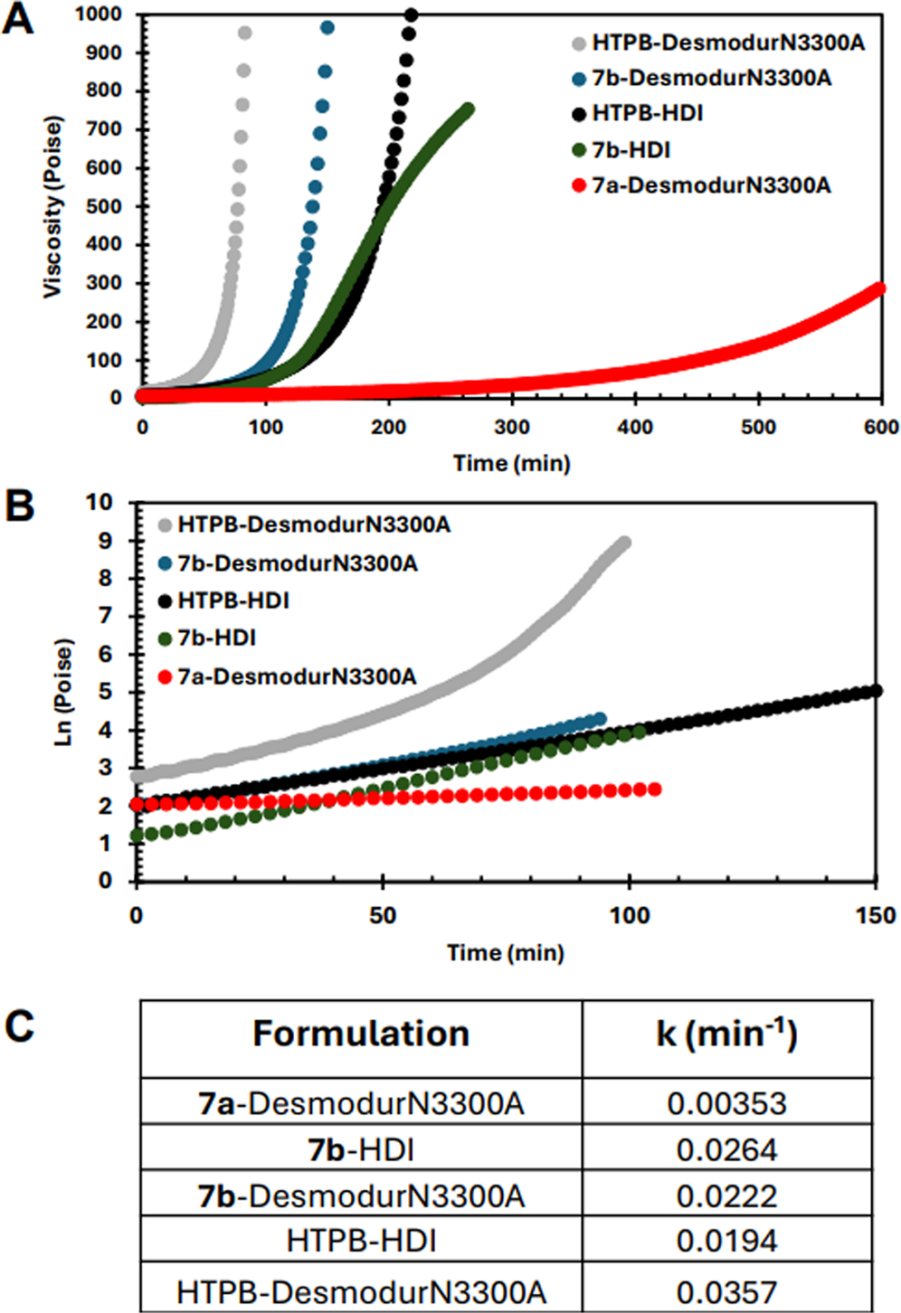
(A) Viscosity measurements of cure over time measured by oscillatory rheology at 60 °C (Details: Viscosity profiles were obtained for kinetics using ETC disposable 25 mm stainless steel plates in parallel geometry at 60 °C using 500 μm gap size. Experiments were conducted as long as 12–16 h. The steady-state shear rate was set to 10 Hz, and data acquisition was set for 2 to 5 min depending on the length of the experiment). (B) Semilog plot of viscosity measurements of cure over time measured by oscillatory rheology at 60 °C. (C) Rate constants for cures as determined from semilog plots from 0 to 60 min.

We next turned to star polymers **7a**–**b** to perform curing experiments with HDI and Desmodur N3300A, measuring the viscosity over time at 60 °C. The results are shown in Figure 4 as the buildup of viscosity over time (Figure 4A) and the semilog plot of the same (Figure 4B). The rate constants are determined from the slopes of the semilog plots from 0 to 60 min and are summarized as well (Figure 4C). In comparing the change in viscosity of the 2-arm star **7a** and 3-arm star **7b** with trifunctional isocyanate Desmodur N3300A, it is apparent that the viscosity buildup of **7b** is an order of magnitude higher than that of **7a**. This is unsurprising as the addition of another hydroxyl group capable of reacting with an isocyanate leads to more cross-linking and therefore higher viscosity. Thus, it can be easily and for the first time conclusively inferred that an increase in the amount of hydroxyl group content leads to a marked decrease in pot-life, which is undesirable for binders. Previous studies on this matter relied on comparisons of materials of different molar masses to make these assumptions, which also contained polymers impure with regard to the number of hydroxyl groups. Viscosity buildup for **7b** with Desmodur N3300A was slower than commercially obtained HTPB. This could be due to the presence of even higher amounts of hydroxyl-group functionality in commercial HTPB, which has on average 2.4–2.6 functionalities per chain, but is not limited to only 2 or 3. Alternatively, this difference could be due to the fact that **7b** contains only trifunctionalized macromolecules, making it more difficult for the third functionality to diffuse to and react with an isocyanate. This hypothesis is more likely, as kinetic experiments revealed (vide infra). We performed differential scanning calorimetry (DSC) on the resulting cures and found that the addition of a hydroxyl group leads to a lowering of the glass transition temperature (*T*_g_). The *T*_g_ values measured were −67.22 °C, −72.24 °C, and −74.00 °C for the cures of Desmodur N3300A with **7a**, commercial HTPB, and **7b**, respectively. Thus, the benefit of a longer pot-life with a purely linear HTPB is offset by an increase in *T*_g_. Further, comparing curing agents, we observed that curing **7b** with HDI also extended the pot-life as compared to Desmodur N3300A, which is again unsurprising given the lesser amount of isocyanates available for reaction.

We next explored the curing of these systems by FTIR, monitoring the strong isocyanate stretch at ∼2270 cm^–1^ as the reaction progressed. These experiments were done to a static mixture of prepolymer and isocyanate, as opposed to rheology experiments which involved steady flow shear. A representative series of spectra for the curing of **7b** with Desmodur N3300A is shown in Figure 5A,B, while the kinetics plot and rate constants are shown in parts C and D. Again, HTPB reacted faster with Desmodur N3300A than HDI, which can be attributed to the presence of more isocyanates available for reaction. The fastest kinetics and therefore the shortest pot-life were that of **7b** and HDI by an order of magnitude. The small-molecule, low-viscosity HDI was able to efficiently diffuse and allow for increased reactivity nearly an order of magnitude greater than HTPB-HDI. Increasing the isocyanate functionality as in Desmodur N3300 and reacting it with **7b** displayed a reaction rate four times slower than the HTPB-Desmodur N3300A formulation, supporting the argument above that with a purely trifunctional macromolecule, hydroxyl and isocyanate groups find it difficult to migrate in the growing network in order to react. This explains why a purely trifunctional polymer has a longer pot-life than one with lower functionality on average, such as commercially available HTPB. To further support this, we also see that bifunctional **7a** reacts faster than **7b** with Desmodur N3300A, as it has solely 2 hydroxyl-groups, and is thus not as restricted as trifunctional **7a**. However, the pot-life for this system is the longest given the lack of urethane linkages available, thereby strongly and conclusively suggesting for the first time that limiting the amount of hydroxyl groups in HTPB is highly desirable for increasing the pot-life. Further, we clearly demonstrate for the first time through analysis of both rheology and kinetics using pristine samples that while low prepolymer viscosity and slower urethane linkage formation may generally be viewed as desirable, the situation is more complex. The difunctional **7a** reacts faster than trifunctional **7b** and has a higher initial viscosity; however, due to the structure of the resulting network, it demonstrates the longest pot-life.

**Figure 5:**
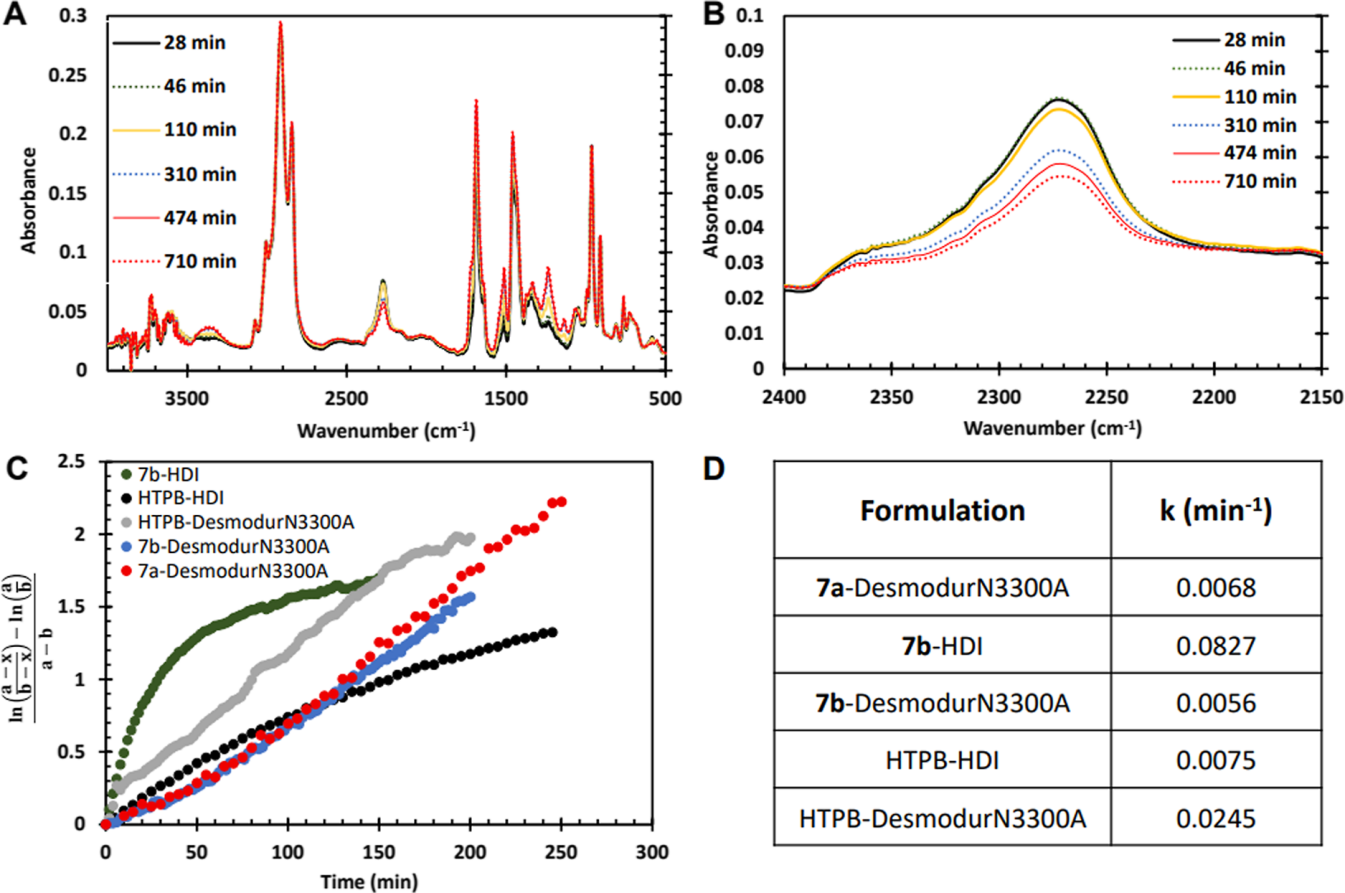
(A) IR spectra at different times for the reaction of **7b** and Desmodur N3300A. (B) Isocyanate region of IR spectra at different times for the reaction of **7b** and Desmodur N3300A. (C) Kinetics plot of curing for all polymer/isocyanate systems, where *a* = initial concentration of hydroxyl, *b* = initial concentration of isocyanate, and *x* = fraction of isocyanate remaining.^2^ (D) Table of rate constants for curing reactions.

## Conclusions

HTPB has played a crucial role as a binder prepolymer for 60 years and will continue to do so for the foreseeable future. Knowledge about how to extend pot-life or improve other materials properties has been limited to reliance on materials that are not truly comparable and/or are structurally impure (e.g., hydroxyl group content). Here, for the first time, we have shown conclusively the effect of the hydroxyl group content on both pot-life and curing kinetics. Specifically, the pot-life decreases as hydroxyl group content increases, and counterintuitively, the rate of urethane formation also slows due to difficulties in migration in the viscous solution. Thus, despite the fact that polymers with additional branching give lower initial viscosities and slower curing kinetics, in order to increase pot-life hydroxyl group content must be curtailed. This is welcomed knowledge, as decreased branching has been shown to lead to longer elongation at break, which is also favorable for binders in SRMs.^2^ This has become more feasible since the advent of HTPB as controlled polymerization technologies has improved greatly. Indeed, in this paper, we have also demonstrated the use of controlled polymerization for butadiene in solution, one of only a handful of examples for this monomer.^28^ Control of end-groups was challenging and required a decrease in the amount of exogenous initiator. Recent advancements in RAFT polymerization have been reported in which initiation occurs without an exogenous initiator (e.g., photoinerferter),^26^ or an initiator that does not become part of the polymer structure.^25^ It should be noted that while the pendant vinyl groups present in the polymers reported herein were not reactive, as in the case of the uncontrolled radical polymerization used traditionally to prepare HTPB, they were generated nonetheless. This is noteworthy, as pendant vinyl groups may affect thermal properties, aging, and viscosity. Work to prepare even more controlled architectures for HTPB in larger scale and by other methods is ongoing and will be reported in due course, and we further encourage others to explore scalable routes to HTPB to better improve its performance.

## Experimental Section

### General Details

2-Bromomethylphenylacetic acid was purchased from Synthonix and used as received. Borane-dimethylsulfide complex, carbon disulfide, 3-mercaptopropionic acid, triethylamine, 4-DMAP, *tert*-butylmethylsilyl chloride, potassium phosphate monohydrate, dicumylperoxide, AIBN, tris­(2-aminoethyl)­amine, and DCC were purchased from Sigma-Aldrich and used as received. HDI was purchased from Sigma-Aldrich and used as received. Hydroxyl-terminated polybutadiene was purchased from Rocket Motor Components. The trifunctional isocyanate Desmodur N3300A was received from Covestro and used as received. All solvents, acids, and other reagents were used as received except where indicated. Except where indicated, reactions were performed under air. NMR spectra were recorded on a JEOL 400 MHz spectrometer. All spectra are referenced to the residual solvent signal. Chloroform-*d* and DMSO-*d*_6_ were purchased from Sigma-Aldrich and used as received. IR spectra of **7a** and **7b** were obtained on a Thermo Nicolet iS10 instrument in the attenuated total reflectance mode. SEC was used to determine molar mass and MMD. An Agilent Technologies 2000 Series system with two Polymer Laboratories PL gel 5 μm 500 Å 300 × 7.5 mm columns and a Wyatt Optilab RI detector was run under the following conditions: THF eluent with a BHT stabilizer and a flow rate of 1.00 mL/min, a temperature of 25 °C, and a nominal sample concentration of 1 mg/mL. To calibrate the SEC system, a set of five narrow dispersity polystyrene standards was used. Polymerizations were performed in a stainless-steel Parr pressure reactor (330 mL volume) with magnetic stirring and a temperature controller. FTIR-ATR Kinetic experiments were conducted on a Thermo Fisher Scientific Nicolet iS50r equipped with Pike GladATR 300 attachment. Kinetic experiments were conducted using time-correlated FTIR-ATR spectroscopy in the range from 500 cm^–1^ to 4500 cm^–1^. The extent of cure of the rheological samples and the kinetic FTIR samples was accomplished qualitatively by assessing the urethane peak at ∼2270 cm^–1^. In order to determine the rate constants from the kinetic data, eq 1 was used. The slope of equation 1 is the rate constant for the second-order reaction of free hydroxyls with isocyanates.^2^

1 $$\frac{\ln\frac{\left(a-x\right)}{\left(b-x\right)}-\ln\left(\frac{a}{b}\right)}{\left(a-b\right)}=kt$$

where *a* is the initial concentration of the hydroxyl compound, *b* is the initial concentration of the isocyanate, *x* is the fraction of NCO functional consumed in the reaction at any given time “*t*”, and *k* is the second-order rate constant.

### Synthesis of New Compounds

#### 2-(4-(Bromomethyl)­phenyl)­ethanol (**2**)

All glassware and syringes were oven-dried before use. A solution of 2-bromomethylphenylacetic acid (**1**, 30.0246 g, 0.1332 mol) in 450 mL of dry tetrahydrofuran (THF, stored over 4 Å molecular sieves) was cooled to 0 °C under nitrogen. Borane–dimethylsulfide complex (19.0 mL, 0.200 mol) was added dropwise with magnetic stirring, with an obvious generation of hydrogen. The solution was then allowed to warm to room temperature and stirred overnight. The solution was cooled to 0 °C, and 1.0 M H_2_SO_4_ was added dropwise to quench the reaction. THF was then removed in vacuo, additional water was added, and the product was extracted in ethyl acetate 3 times. The solution was dried over MgSO_4_ and concentrated. Compound **2** was purified by column chromatography (2:1–1:2 hexane/ethyl acetate), furnishing a white powder (24.0046 g, yield = 85%). Compound **2** was characterized by ^1^H and ^13^C NMR, matching those reported in the literature.^33^ In previous reports, quenching the reaction with water yielded similar yields of **2**; however, in our hands, this failed in repeated attempts, yielding <1% of product. ^1^H (400 MHz, 25 °C, DMSO-*d*_6_) NMR: 7.34 (d, *J* = 8.0 Hz, Ar*H*, 2H), 7.20 (d, *J* = 8.0 Hz, Ar*H*, 2H), 4.68 (s, Br–C*H*_2_–Ar, 2H), 4.57 (br, O*H*, 1H), 3.59 (t, *J* = 7.0 Hz, HO–C*H*_2_–CH_2_–Ar, 2H), 2.71 (t, *J* = 7.0 Hz, HO–CH_2_–C*H*_2_–Ar, 2H).
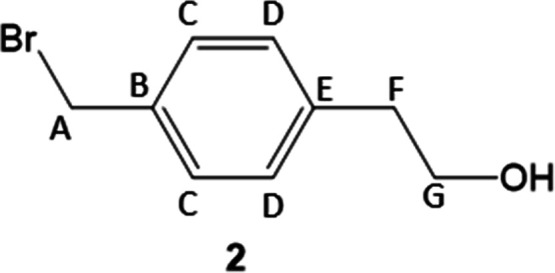


^13^C (100 MHz, 25 °C, DMSO-*d*_6_) NMR: 140.0 (E), 135.5 (B), 129.2 (C), 129.2 (D), 62.0 (G), 38.7 (F), 34.8 (A).

#### (2-(4­(Bromomethyl)­phenyl)­ethoxy)-*tert*-butyldimethylsilane (**3**)

All glassware was oven-dried before use. Compound **2** (4.0841 g, 0.0190 mol) was dissolved in 200 mL of dry dichloromethane (DCM, stored over 4 Å molecular sieves). *Tert*-Butyldimethylsilyl chloride (3.5228 g, 0.0234 mol), triethylamine (3.28 mL, 0.0234 mol, stored over 4 Å molecular sieves), and DMAP (0.1092 g, 0.0009 mol) were quickly added, and the reaction mixture was allowed to stir overnight at room temperature. The solution was then washed with saturated NH_4_Cl. The aqueous layer was extracted with DCM. The combined organic layers were then washed with water 3 times, dried over MgSO_4_, and concentrated. Compound **3** was purified by column chromatography (1:9 ethyl acetate/hexane) to yield a tan oil (1.5280 g, yield = 24%). ^1^H (400 MHz, 25 °C, chloroform-*d*) NMR: 7.30 (d, *J* = 8.1 Hz, Ar*H*, 2H), 7.20 (d, *J* = 8.1 Hz, Ar*H*, 2H), 4.57 (s, Br–C*H*_2_–Ar, 2H), 3.79 (t, *J* = 7.0 Hz, TBSO–C*H*_2_–CH_2_–Ar, 2H), 2.82 (t, *J* = 7.0 Hz, TBSO–CH_2_–C*H*_2_–Ar, 2H), 0.87 (s, Si­(CH_3_)_2_C­(C*H*_3_)_3_, 9H), −0.02 (s, Si­(C*H*_3_)_2_C­(CH_3_)_3_, 6H).
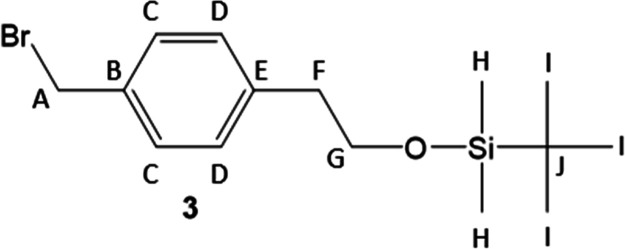


^13^C (100 MHz, 25 °C, chloroform-*d*) NMR: 139.8 (E), 135.5 (B), 129.7 (C), 128.7 (D), 64.5 (G), 46.4 (F), 39.4 (A), 26.1 (I), 18.5 (J), −5.3 (H). Note: In scaling up the synthesis of **3**, significant amounts of the symmetric TBS silyl ether are obtained and difficult to remove by column chromatography. The amount of this impurity may be determined by ^1^H NMR and carried forward to be removed in the following synthesis with no impact on the yield.

#### Compound **4**

3-Mercaptopropionic acid (1.1227 g, 0.0106 mol) in 10 mL of acetone was added to potassium phosphate monohydrate (2.4322 g, 0.0106 mol) as a suspension in 40 mL of acetone and stirred vigorously for 10 min. Carbon disulfide (1.82 mL, 0.0314 mol) was added, and the solution was stirred vigorously for 10 min, during which time the solution turned yellow. Compound **3** (3.4667 g, 0.0105 mol, containing TBS_2_O impurity of known quantity which was accounted for) was added, and the solution was heated to 40 °C and stirred overnight. Volatiles were removed in vacuo, and the mixture was dissolved in ethyl acetate and washed with a saturated brine. The aqueous layer was extracted with ethyl acetate 3 more times and dried over MgSO_4_. The solution was concentrated, and **4** was purified by column chromatography (1:9 ethyl acetate/hexane to 100% ethyl acetate) to yield a yellow oil (4.1601 g, yield = 90%). Note: A small TBS_2_O impurity remains in some syntheses and is easily accounted for in terms of the yield by ^1^H NMR analysis. It is easily removed in a subsequent step. ^1^H (400 MHz, 25 °C, chloroform-*d*) NMR: 7.24 (d, *J* = 8.1 Hz, Ar*H*, 2H), 7.15 (d, *J* = 8.1 Hz, Ar*H*, 2H) 4.57 (s, Ar–C*H*_2_–S, 2H), 3.78 (t, *J* = 7.0 Hz, Ar–CH_2_–C*H*_2_–OTBS, 2H), 3.61 (t, *J* = 7.0 Hz, C­(=O)–CH_2_–C*H*_2_–S, 2H), 2.76–2.87 (m, C­(=O)–C*H*_2_–CH_2_–S, Ar–C*H*_2_–CH_2_–OTBS, 4H), 0.86 (s, Si­(CH_3_)_2_C­(C*H*_3_)_3_, 9H), −0.02 (s, Si­(C*H*_3_)_2_C­(CH_3_)_3_, 6H).
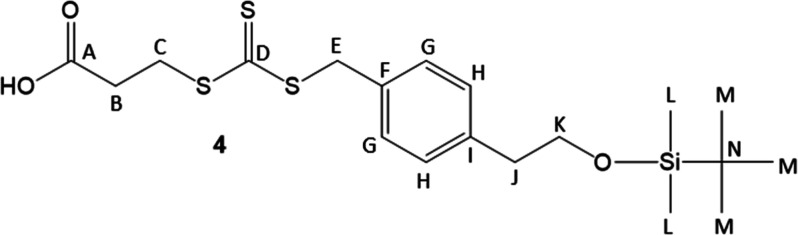


^13^C (100 MHz, 25 °C, chloroform-*d*) NMR: 223.0 (D), 177.3 (A), 139.1 (I), 132.5 (F), 129.7 (G), 129.3 (H), 64.5 (K), 41.6 (E), 39.3 (J), 33.1 (C), 31.0 (B), 26.0 (M), 18.5 (N), −5.3 (L). Previously, a similar reaction was reported using unfunctionalized bromobenzene. This procedure has been modified to account for the need for heat and longer reaction times.^34^

#### 2-Arm CTA TBS-Protected Precursor (**5a**)

Compound **4** (1.3407 g, 0.003113 mol) was dissolved in 100 mL of DCM and cooled to 0 °C. DCC (0.7056 g, 0.003419 mol), DMAP (0.0239 g, 0.000196 mol), and diaminoethane (0.0832 g, 0.00138 mol) were added, and the solution was warmed to room temperature and stirred overnight. The solution was cooled to 0 °C, forming a white precipitate. The solution was vacuum-filtered, and volatiles were removed in vacuo. The resulting material was dissolved in carbon tetrachloride at 0 °C and filtered, and the volatiles were again removed in vacuo to yield a yellow waxy solid. Compound **5a** was purified by column chromatography (1:1 ethyl acetate/hexane to 100% ethyl acetate) to yield a yellow waxy solid (1.9806 g, yield = 65%). ^1^H (400 MHz, 25 °C, chloroform-*d*) NMR: 7.23 (d, *J* = 8.0, Ar*H*, 2H), 7.14 (d, *J* = 8.0 Hz, Ar*H*, 2H), 6.32 (br, N*H*, 1H), 4.56 (s, S–C*H*_2_–Ar, 2H), 3.77 (t, *J* = 7.0 Hz, Ar–CH_2_–C*H*_2_–OTBS, 2H), 3.64 (t, *J* = 6.9 Hz, C­(=O)–CH_2_–C*H*_2_–S, 2H), 3.38 (m, NH–C*H*_2_–, 2H), 2.79 (t, *J* = 7.0 Hz, Ar–C*H*_2_–CH_2_–OTBS, 2H), 2.63 (t, *J* = 6.9 Hz, C­(=O)–C*H*_2_–CH_2_–S, 2H), 0.86 (s, Si­(CH_3_)_2_C­(C*H*_3_)_3_, 9H), −0.03 (2, Si­(C*H*_3_)_2_C­(CH_3_)_3_, 6H).
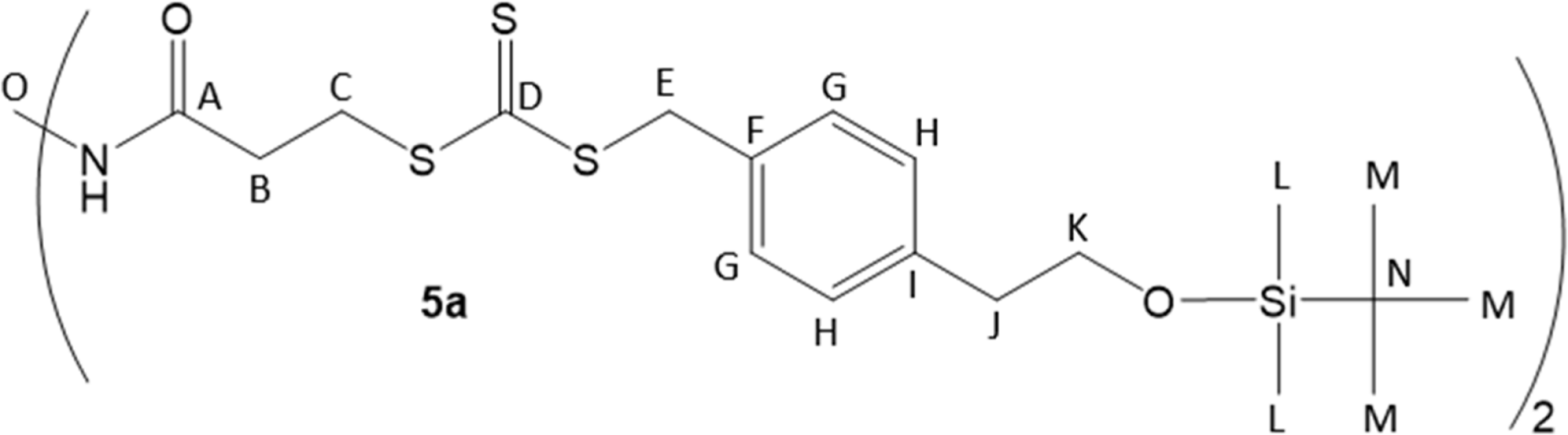


^13^C (100 MHz, 25 °C, chloroform-*d*) NMR: 223.4 (D), 171.7 (A), 139.1 (I), 132.5 (F), 129.7 (G), 129.3 (H), 64.5 (K), 41.6 (E), 40.1 (J), 39.4 (O), 35.0 (C), 32.2 (B), 26.0 (M), 18.5 (N), −5.3 (L).

#### 3-Arm CTA TBS-Protected Precursor (**5b**)

Compound **4** (3.9622 g, 0.009199 mol) was dissolved in DCM and cooled to 0 °C. DCC (2.1131 g, 0.010241 mol), DMAP (0.0632 g, 0.000517 mol), and tris­(2-aminoethyl)­amine (0.4015 g, 0.002745 mol) were added, and the solution was warmed to room temperature and stirred overnight. The solution was cooled to 0 °C, forming a white precipitate. The solution was vacuum-filtered, and volatiles were removed in vacuo. The resulting oil was dissolved in carbon tetrachloride at 0 °C and filtered, and volatiles were again removed in vacuo. Compound **5b** was purified by column chromatography (1:1 ethyl acetate/hexane to 100% ethyl acetate). Note: the product elutes with the 100% ethyl acetate, but significant volumes are required to retrieve all product. Volatiles were removed in vacuo to furnish **5b** as a viscous yellow oil (2.6181 g, yield = 62%). ^1^H (400 MHz, 25 °C, chloroform-*d*) NMR: 7.22 (d, *J* = 8.0 Hz, Ar*H*, 2H), 7.13 (d, *J* = 8.0 Hz, Ar*H*, 2H), 6.65 (t, *J* = 5.4 Hz, N*H*, 1H), 4.55 (s, S–C*H*_2_–Ar, 2H), 3.76 (t, *J* = 7.0 Hz, Ar–CH_2_–C*H*_2_–OTBS, 2H), 3.63 (t, *J* = 6.9 Hz, C­(=O)–CH_2_–C*H*_2_–S, 2H), 3.25 (q, *J* = 5.4 Hz, N–CH_2_–C*H*_2_–NH, 2H), 2.78 (t, *J* = 7.0 Hz, Ar–C*H*_2_–CH_2_–OTBS, 2H), 2.64 (t, *J* = 6.9 Hz, C­(=O)–C*H*_2_–CH_2_–S, 2H), 2.52 (br, N–C*H*_2_–CH_2_–NH, 2H), 0.86 (s, Si­(CH_3_)_2_C­(C*H*_3_)_3_, 9H), −0.03 (s, Si­(C*H*_3_)_2_C­(CH_3_)_3_, 6H).
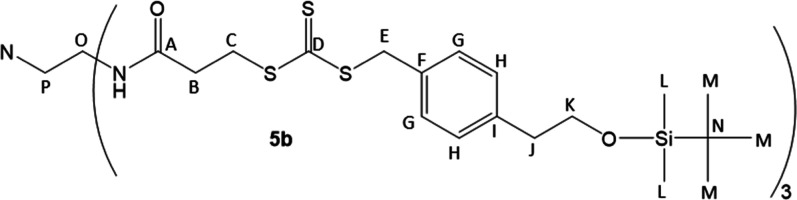


^13^C (100 MHz, 25 °C, chloroform-*d*) NMR: 223.6 (D), 171.3 (A), 139.0 (I), 132.5 (F), 129.7 (G), 129.3 (H), 64.5 (K), 54.8 (P), 41.6 (E), 39.4 (O), 38.2 (J), 34.9 (C), 32.4 (B), 26.0 (M), 18.5 (N), −5.3 (L).

#### 2-Arm CTA (**6a**)

Compound **5a** (1.9606 g, 0.00221 mol) was dissolved in 100 mL of THF. 44 mL of 1.0 M HCl was added dropwise, and the solution was stirred overnight at room temperature. Saturated NaHCO_3_ was added, and the product was extracted in ethyl acetate 3 times. After drying over MgSO_4_, volatiles were removed in vacuo*.* The crude material was washed on a fritted filter funnel packed with silica gel with DCM, ethyl acetate, and methanol before being eluted with DMSO. DMSO was removed by adding ethyl acetate and washing with water several times, and leaving under high vacuum for 2 days, to provide **6a** as a yellow powder (0.9605 g, yield = 66%) ^1^H (400 MHz, 25 °C, DMSO-*d*_6_) NMR: 7.98 (br, N*H*, 1H), 7.27 (d, *J* = 8.1 Hz, Ar*H*, 2H), 7.17 (d, *J* = 8.1 Hz, Ar*H*, 2H), 4.65 (t, *J* = 5.3 Hz, O*H*, 1H), 4.62 (s, Ar–C*H*_2_–S, 2H), 3.50–3.60 (m, NH–C*H*_2_–, Ar–CH_2_–C*H*_2_–OH, 4H), 3.08 (m, S–C*H*_2_–CH_2_–C­(=O), 2H), 2.68 (t, *J* = 7.0 Hz, Ar–C*H*_2_–CH_2_–OH, 2H), 2.52 (t, *J* = 6.9 Hz, S–CH_2_–C*H*_2_–C­(=O), 2H).
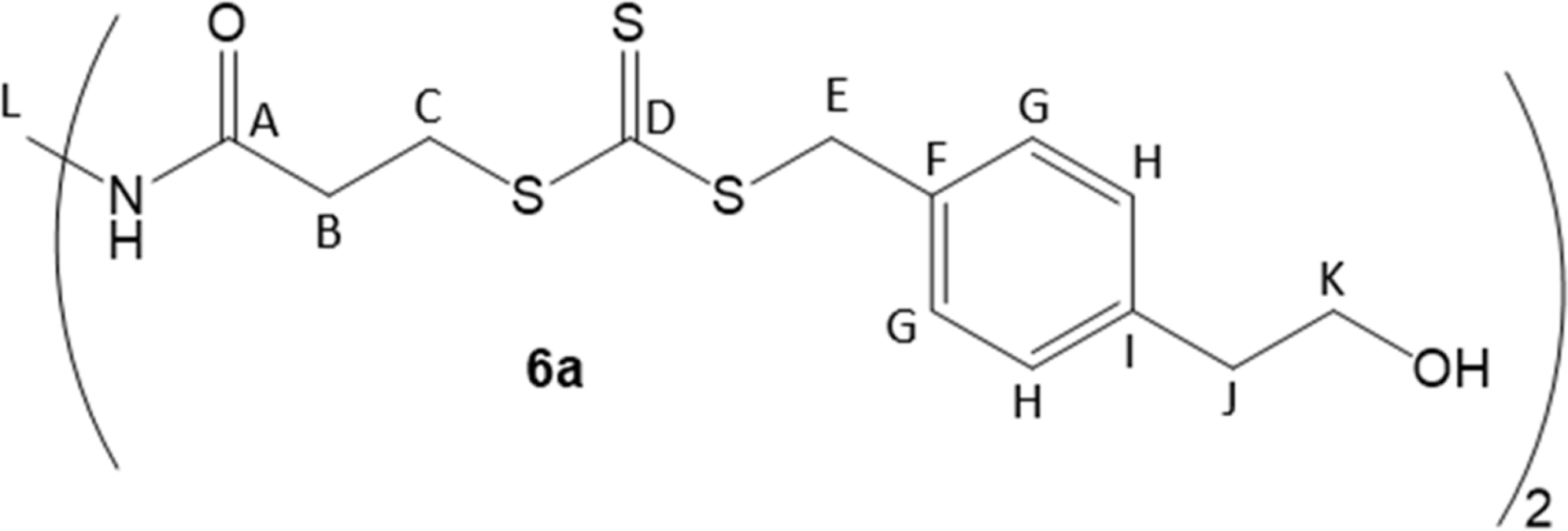


^13^C (100 MHz, 25 °C, DMSO-*d*_6_) NMR: 223.7 (D), 169.8 (A), 139.2 (I), 132.5 (F), 129.2 (G), 129.1 (H), 62.0 (K), 40.2 (E), 38.3 (J), 38.3 (L), 33.4 (C), 32.3 (B). IR­(ATR): (3302, 2944, 1638, 1050) cm^–1^.

#### 3-Arm CTA (**6b**)

Compound **5b** (0.8828 g, 0.0006377 mol) was dissolved in 50 mL of THF. 13 mL of 1.0 M HCl was added dropwise, and the solution was stirred overnight at room temperature. Saturated NaHCO_3_ was added, and the product was extracted in ethyl acetate 3 times. After drying over MgSO_4_, volatiles were removed in vacuo*.* The crude material was washed on a fritted filter funnel packed with silica gel with ethyl acetate before being eluted with methanol. Volatiles were removed, and compound **6b** was then crystallized at −20 °C from a mixture of methanol and ethyl acetate to furnish a yellow crystalline material (0.4159 g, yield = 63%). ^1^H (400 MHz, 25 °C, DMSO-*d*_6_) NMR: 7.86 (t, *J* = 5.5 Hz, N*H*, 1H), 7.26 (d, *J* = 8.0 Hz, Ar*H*, 2H), 7.16 (d, *J* = 8.0 Hz, Ar*H*, 2H), 4.64 (t, *J* = 4.7 Hz, O*H*, 1H), 4.61 (s, S–C*H*_2_-Ar, 2H), 3.48–3.60 (m, C­(=O)–CH_2_–C*H*_2_–S, Ar–CH_2_–C*H*_2_–OH, 4H), 3.07 (q, *J* = 6.0, N–CH_2_–C*H*_2_–NH, 2H), 2.68 (t, *J* = 7.0 Hz, Ar–C*H*_2_–CH_2_–OH, 2H), 2.54 (t, *J* = 6.9 Hz, C­(=O)–C*H*_2_–CH_2_–S, 2H), 2.45 (t, *J* = 6.3, N–C*H*_2_–CH_2_–NH, 2H).
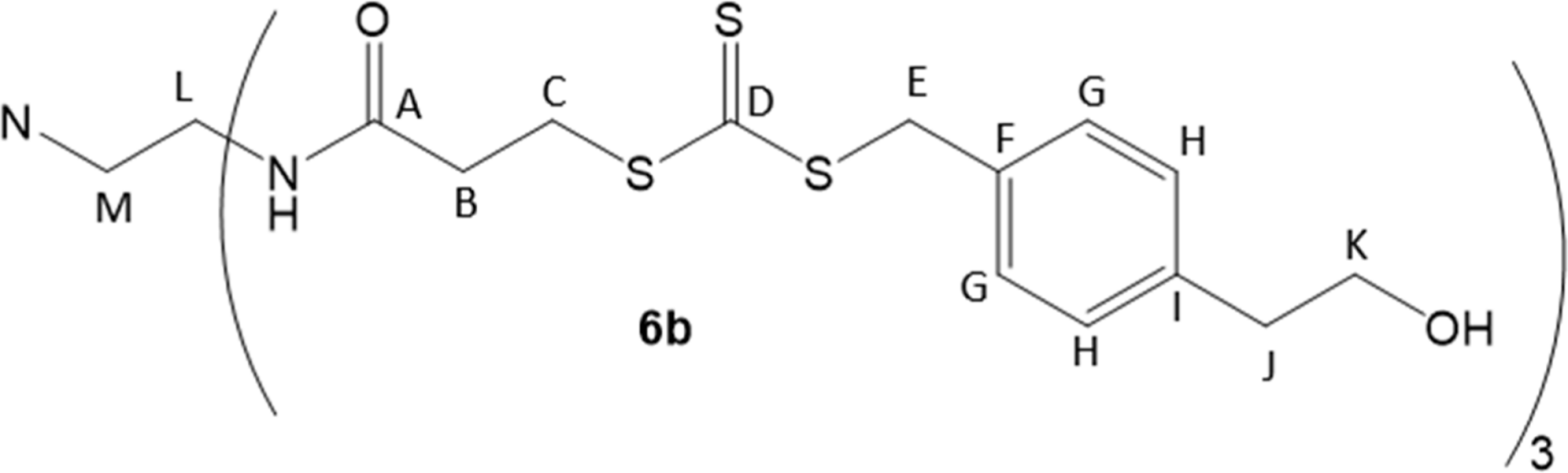


^13^C (100 MHz, 25 °C, DMSO-*d*_6_) NMR: 223.6 (D), 169.8 (A), 139.2 (I), 132.5 (F), 129.3 (G), 129.2 (H), 62.1 (K), 53.6 (M), 40.3 (E), 38.7 (L), 37.2 (J), 33.4 (C), 32.5 (B). IR­(ATR): 3325, 2944, 1641, 1066 cm^–1^.

## Polymerizations

A representative procedure is given. Others may vary in the amounts of reactants or reaction time. All reactions were performed with 80 mL of total solvent. **6a** (0.1019 g, 0.000159 mol) and DCP (0.0043 g, 0.0000159 mol) were dissolved in 55 mL of toluene and 25 mL of DMSO in the Parr reactor, equipped with a magnetic stir bar. The chamber was connected to the reactor head, and the head space was evacuated and backfilled with nitrogen. The chamber was purged with nitrogen for 5 min and then evacuated again. To the evacuated chamber, butadiene was added until a pressure of 16 psi was achieved, at which point the chamber was quickly sealed. If the chamber is not sealed quickly, butadiene will continue to dissolve into the degassed solution, resulting in very high molar mass polymers. Upon adding butadiene, the temperature increased by 2–3 °C. The sealed chamber was then heated to 120 °C for 6 days. After the reaction was completed, it was cooled to room temperature and opened. Toluene was removed in vacuo. Ethyl acetate was added and washed with water several times to remove DMSO. The organic layer was dried over MgSO_4_, and the mixture was concentrated in vacuo. 0.9 mg portion of BHT was added to prevent cross-linking, and the remaining volatiles were removed in vacuo to furnish a yellow, viscous polymer (0.4060 g) which was stored in the freezer to prevent cross-linking. Representative NMR and IR spectra are provided in the Supporting Information.^35^

## Measurements

Rheology was conducted on a TA Instruments Discovery Hybrid Rheometer (HR-30) equipped with an ETC heating jacket and a Peltier plate. To determine 25 and 60 °C initial viscosities of the components, the rheometer was equipped with a 40 mm stainless steel cone and late geometry and the Peltier plate, set to the necessary temperature, a shear sweep was conducted from 0.01 to 100.0 1/s and from 100.0 to 0.01 1/s where in five data points per decade were recorded. In order to ascertain information regarding the gel point, oscillatory experiments were conducted. Gel point measurements were conducted in parallel plate geometry with 25 mm stainless steel plates employing a 500 μm gap. The experimental conditions are as follows: Oscillation stress of 5.0 Pa, single-point frequency of 2.0 Hz, and temperature of 60 °C. Initial experiments were conducted up to 16 h. Viscosity profiles were obtained for kinetics using ETC disposable 25 mm stainless steel plates in parallel geometry at 60 °C using a 500 μm gap size. Experiments were conducted as long as 12–16 h. The steady-state shear rate was set to 10 Hz, and data acquisition was set for two-five min depending on the length of the experiment. DSC was performed using a TA Instruments Q800. Samples were analyzed at a scan rate of 10 °C/min from −90 to 60 °C. Samples were evaluated in aluminum hermetically sealed pans for analysis of *T*_g_.

## Supplementary Material



## Notes

NMR spectra, IR spectra, model RAFT of butadiene, summary of model polymerizations, and chromatograms (PDF)
