# Supplementary material for: Synthesis and Properties of Star-Shaped Hydroxyl-Terminated Polybutadiene via RAFT Polymerization: A Model for a Binder Prepolymer
Source: ACS Omega. 2025 Nov 3;10(45):54274–85. doi: 10.1021/acsomega.5c06683 (PMC12631675; doi:10.1021/acsomega.5c06683)
Supplement: Supplementary file 1 [file ao5c06683_si_001.pdf]

Supporting Information

for the article entitled

Synthesis of Star-Shaped Hydroxyl-Terminated Polybutadiene via RAFT Polymerization: A Model for Binder Prepolymer

authored by

Wesley S. Farrell,*,† Edward Gravois,† Nicholas C. Molineaux,† Anthony M. Clay‡

†Chemistry Department, United States Naval Academy, 572M Holloway Road, Annapolis, Maryland 21402, United States

‡DEVCOM – Army Research Lab

*E-mail: wfarrell@usna.edu

NMR Spectra of New Compounds

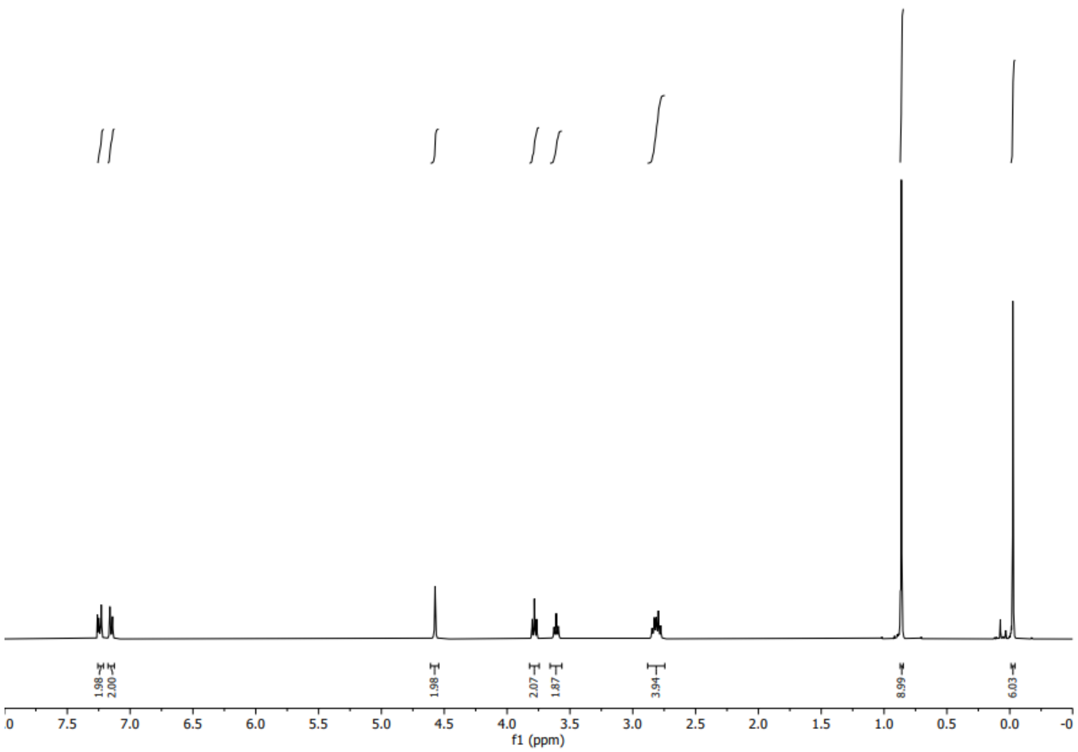

Figure S1. ^{1}H (400 MHz, 25 °C, chloroform-*d*) NMR of compound **4**.

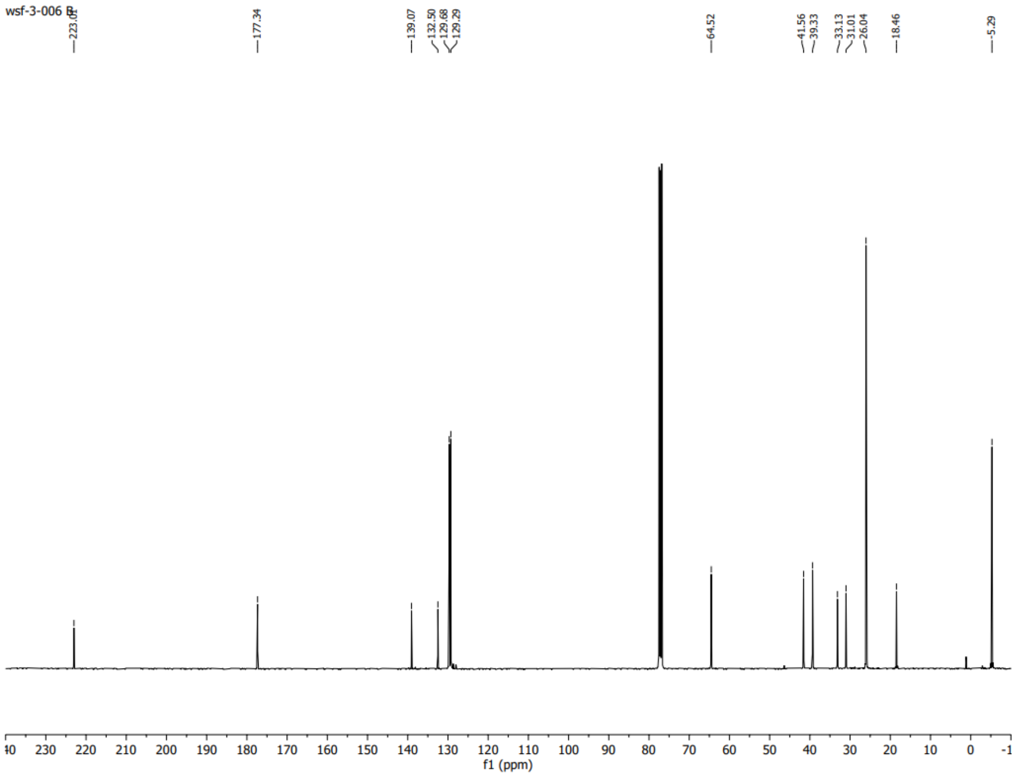

Figure S2. ^{13}C (100 MHz, 25 °C, chloroform-*d*) NMR of compound **4**.

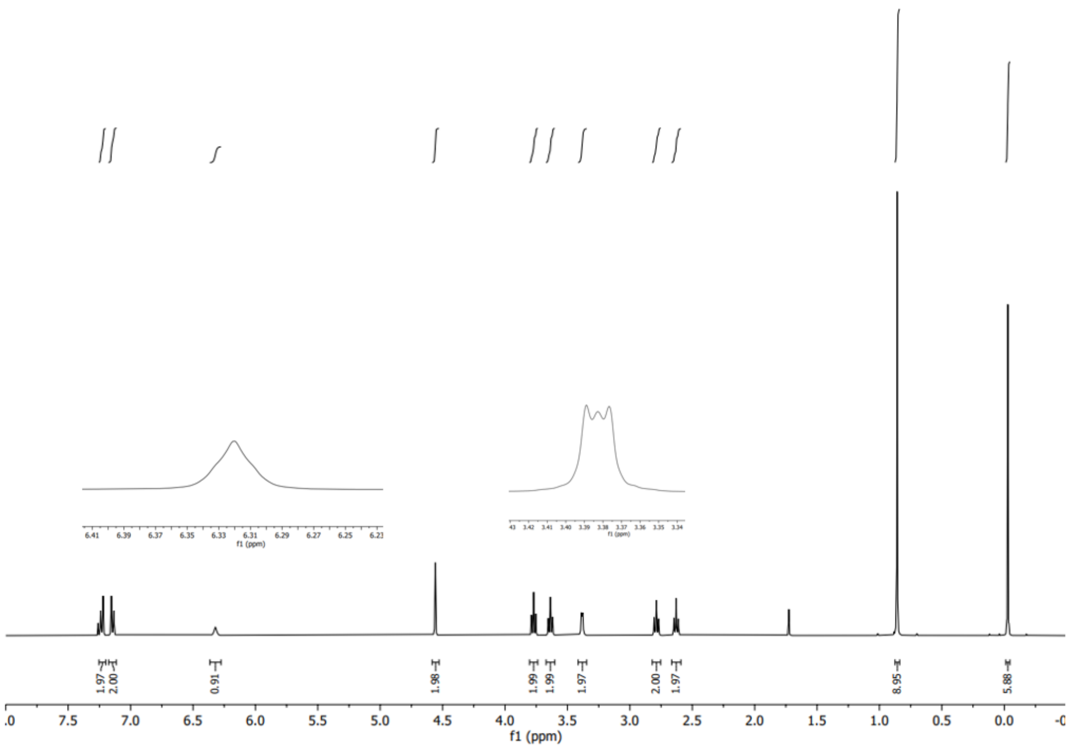

Figure S3. ^{1}H (400 MHz, 25 °C, chloroform-*d*) NMR of compound **5a**.

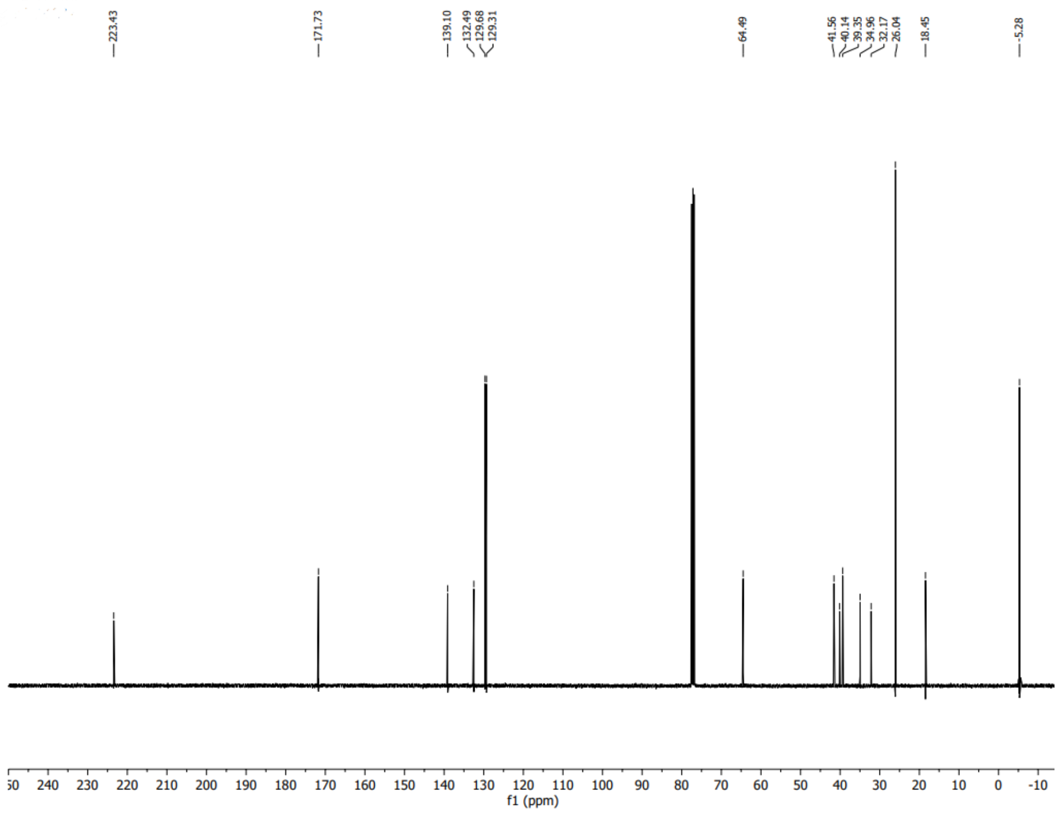

Figure S4. ^{13}C (100 MHz, 25 °C, chloroform-*d*) NMR of compound **5a**.

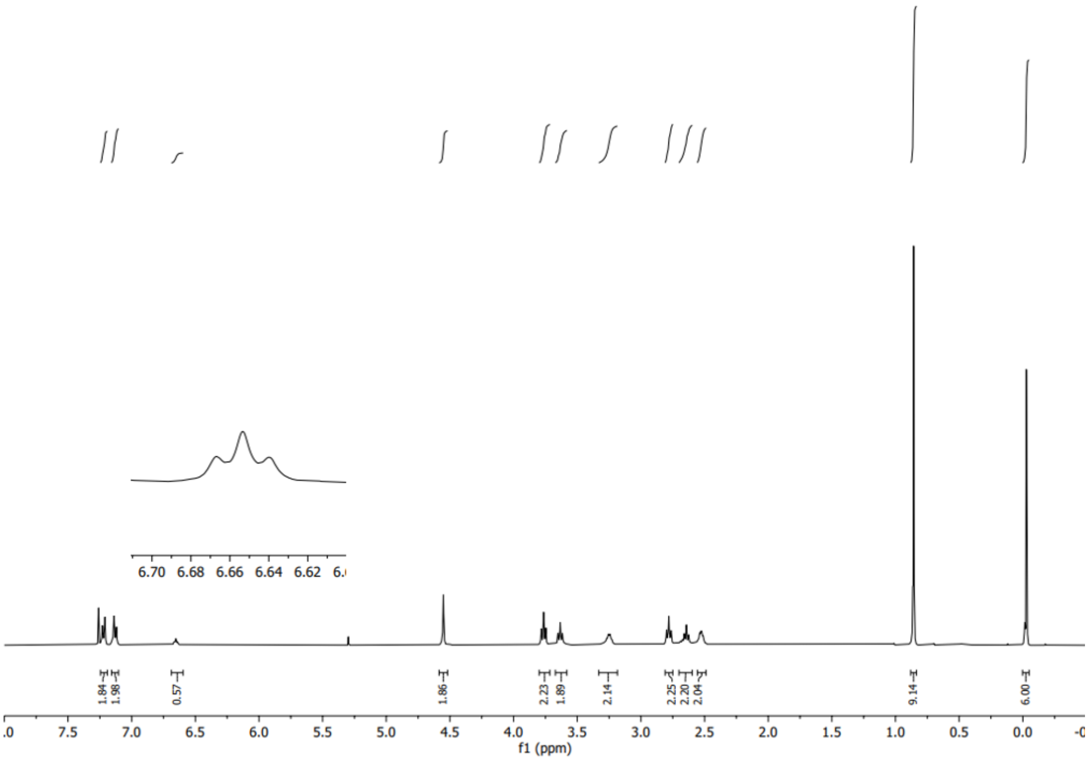

Figure S5. ^{1}H (400 MHz, 25 °C, chloroform-*d*) NMR of compound **5b**.

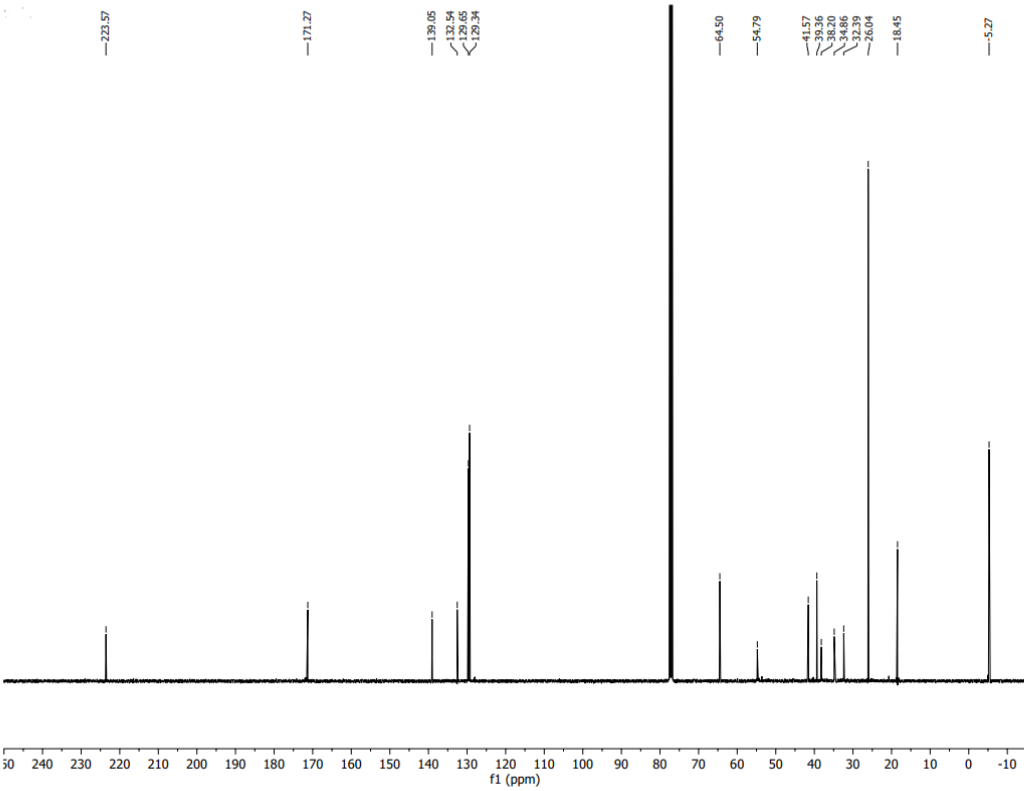

Figure S6. ^{13}C (100 MHz, 25 °C, chloroform-*d*) NMR of compound **5b**.

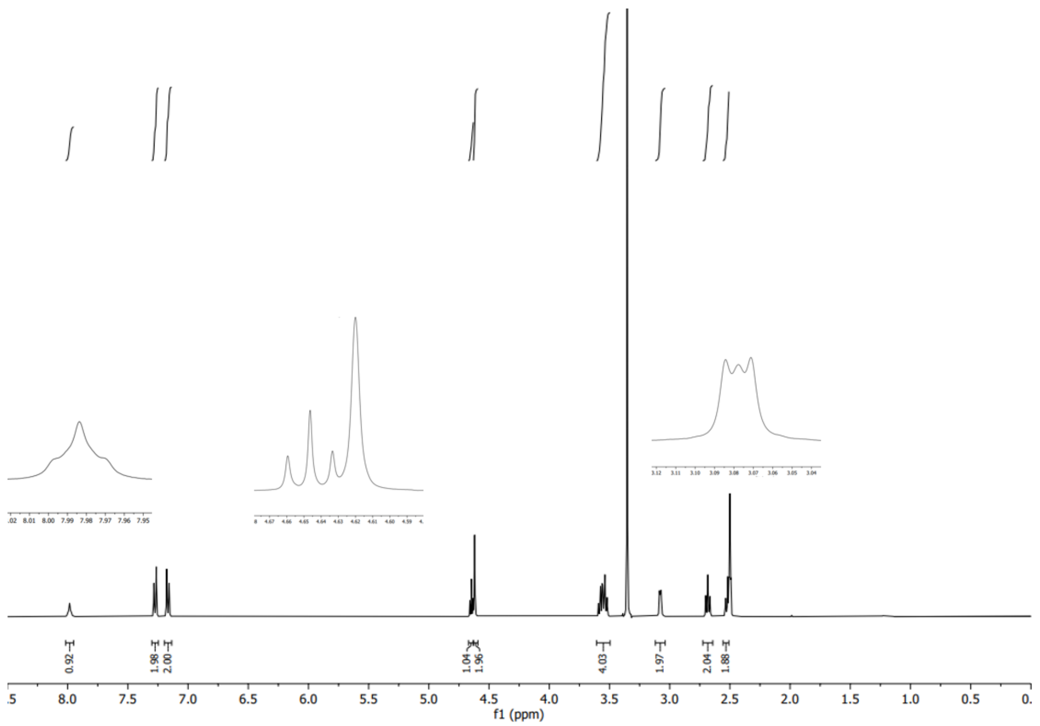

Figure S7. ^{1}H (400 MHz, 25 °C, DMSO-*d*$_6$) NMR of compound **6a**. The large singlet at 3.33 ppm is H_2O.

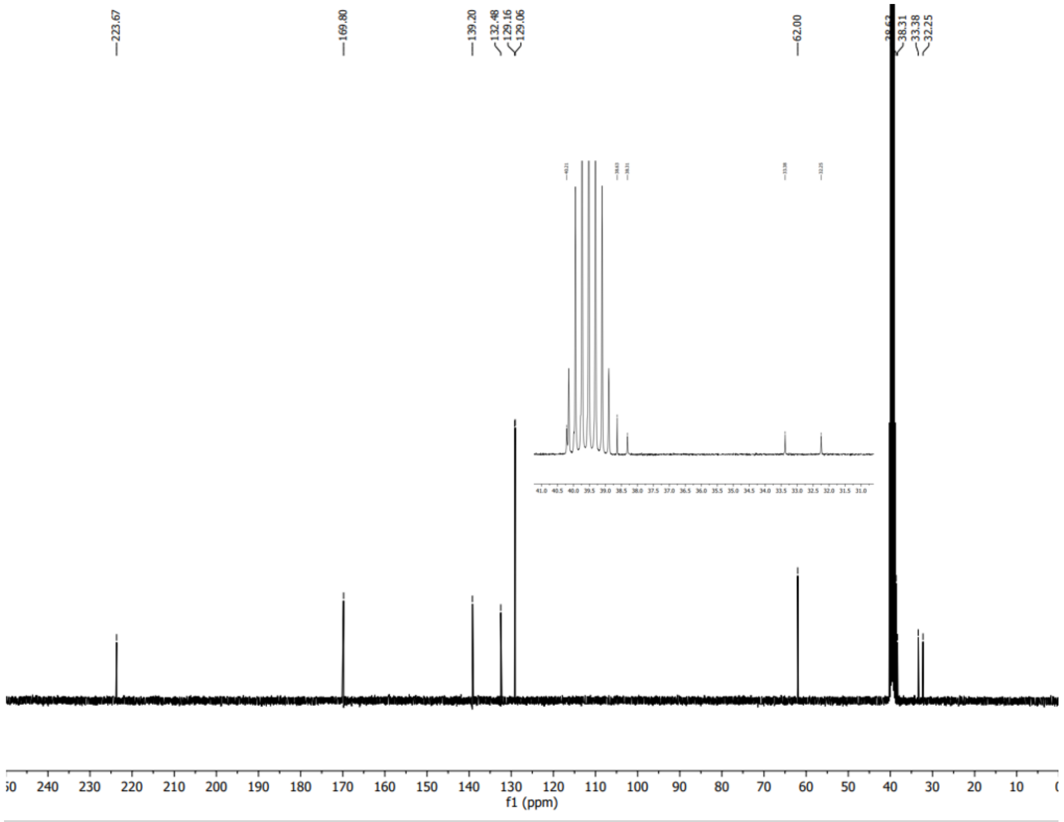

Figure S8. ^{13}C (100 MHz, 25 °C, DMSO-*d*$_6$) NMR of compound **6a**.

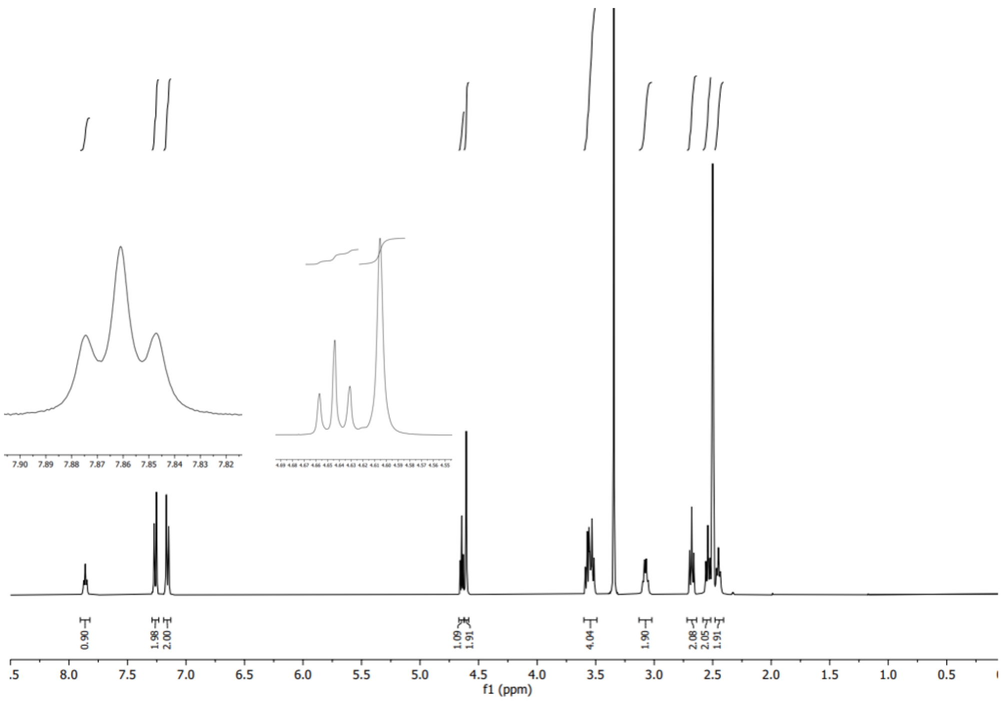

Figure S9. ^{1}H (400 MHz, 25 °C, DMSO-d_6) NMR of compound **6b**. The large singlet at 3.33 ppm is H_2O.

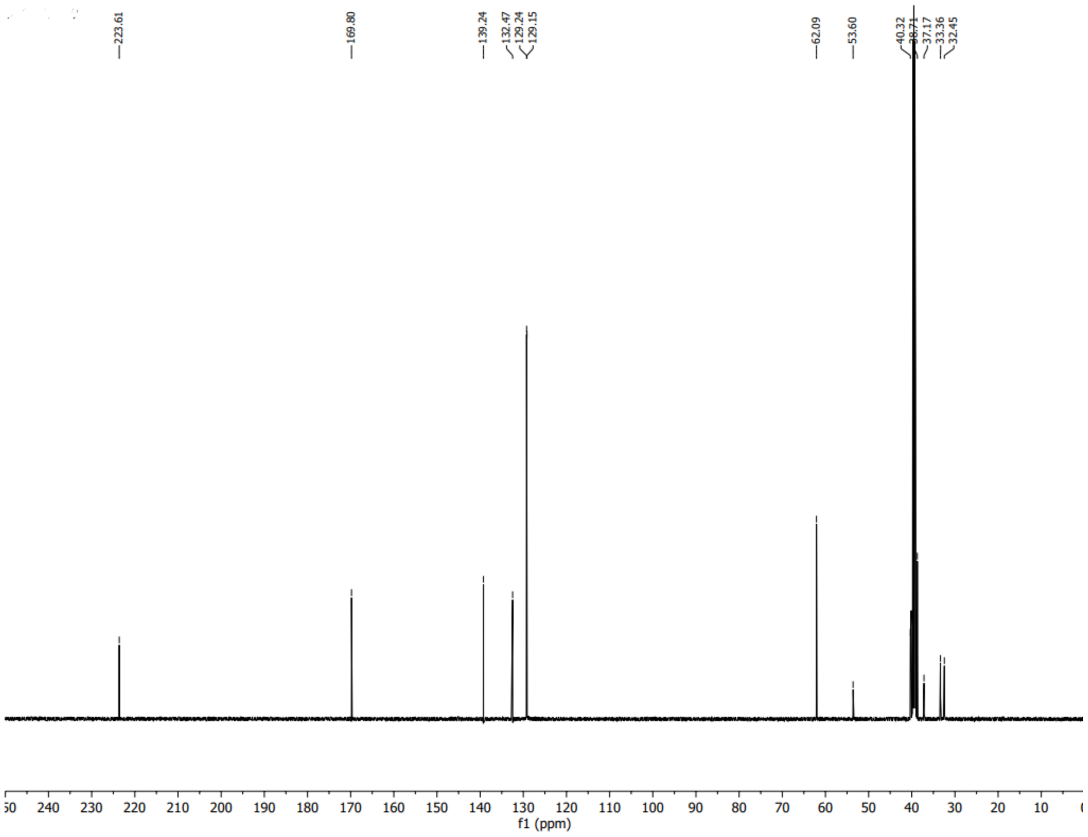

Figure S10. ^{13}C (100 MHz, 25 °C, DMSO-d_6) NMR of compound **6b**.

IR Spectra of CTAs

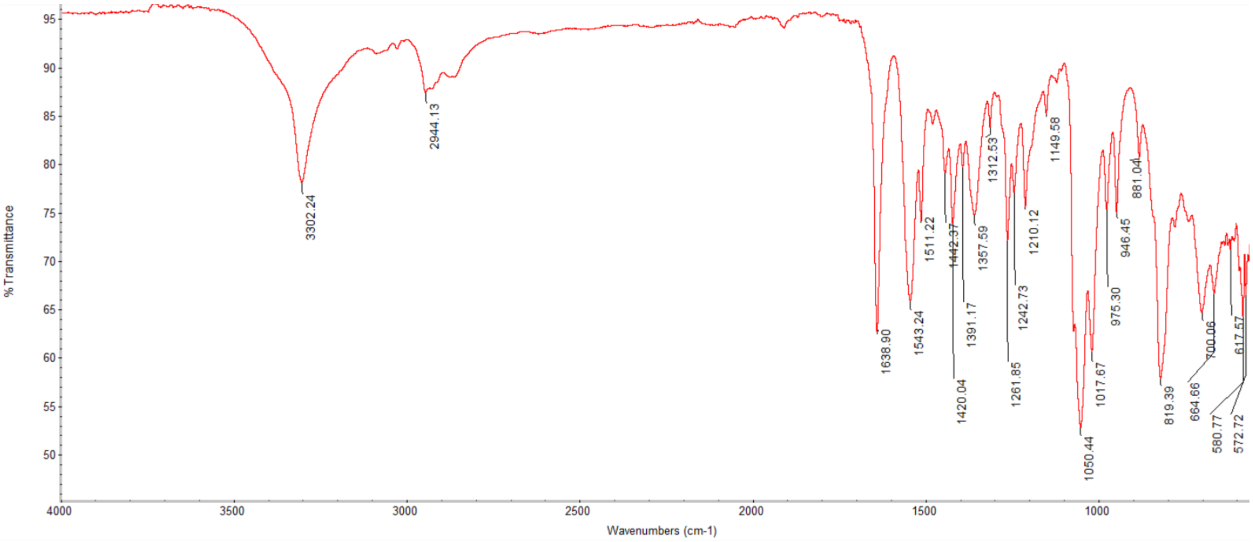

Figure S11. IR(ATR) spectrum of compound **6a**.

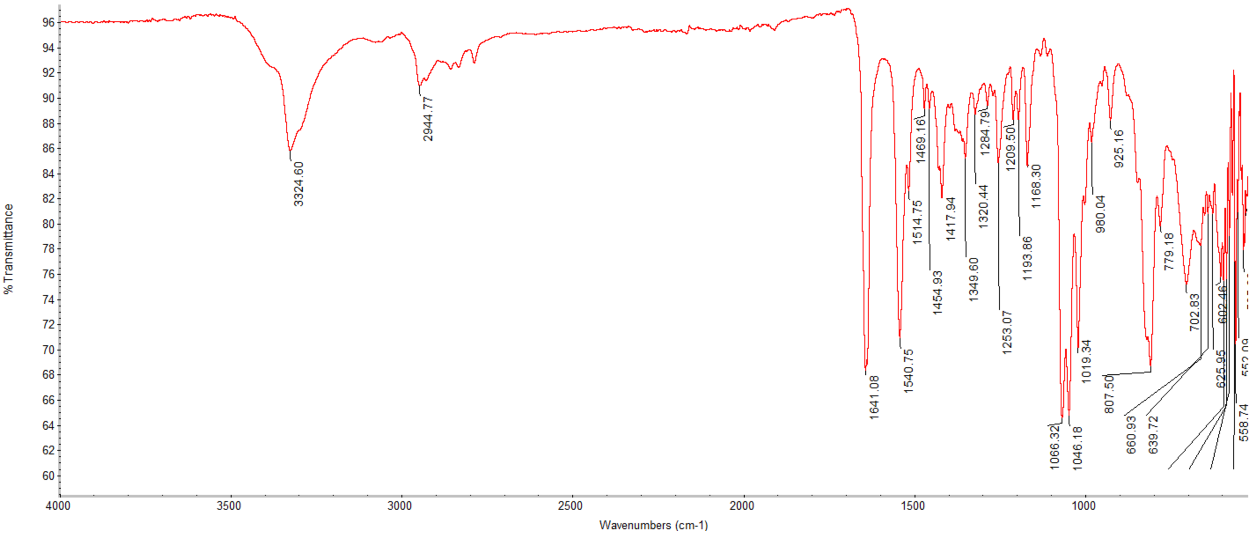

Figure S12. IR(ATR) spectrum of compound **6b**.

NMR of Polymers

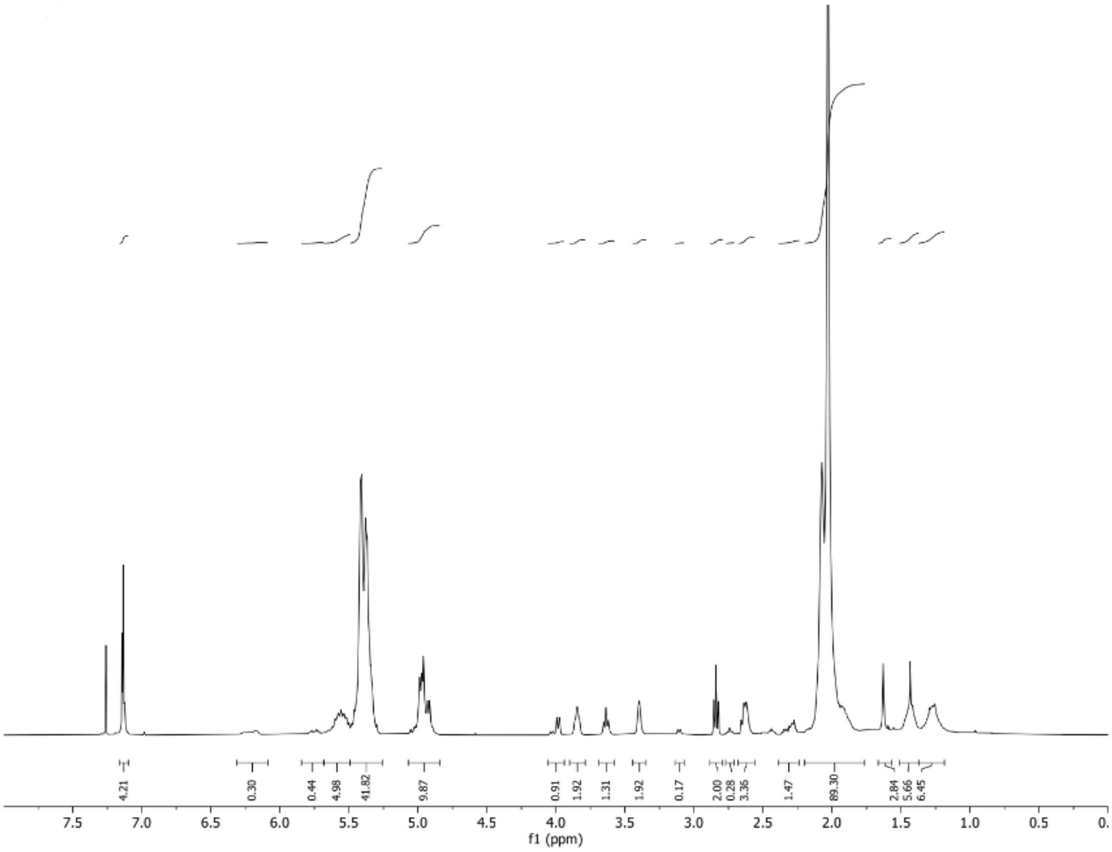

Figure S13. Representative ^{1}H (400 MHz, 25 °C, chloroform-*d*) NMR of 2-arm star polymer (**7a**).

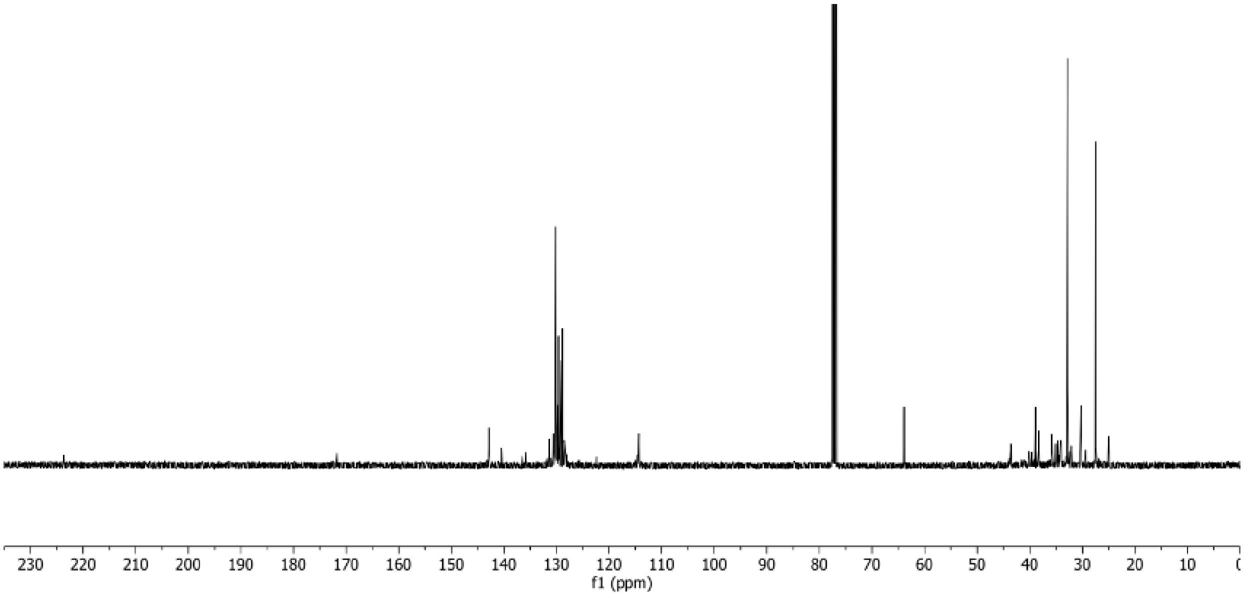

Figure S14. Representative ^{13}C (100 MHz, 25 °C, chloroform-*d*) NMR of **7a**.

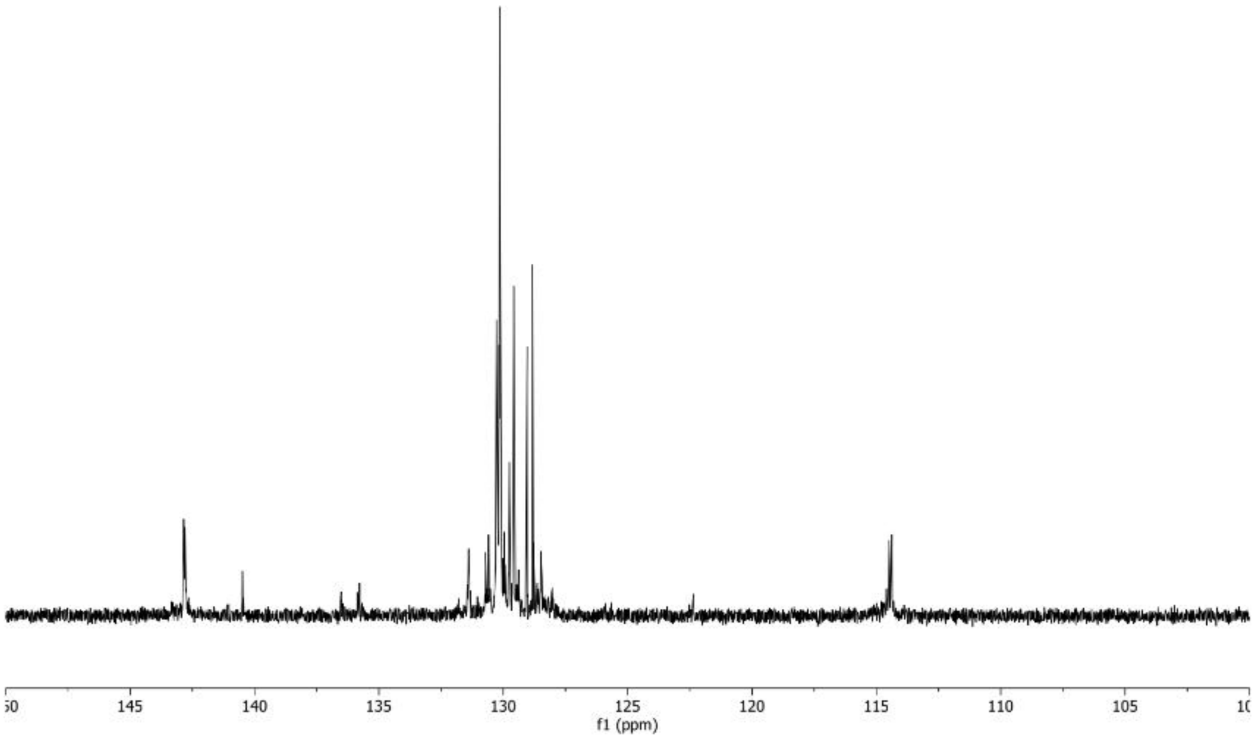

Figure S15. Representative ^{13}C (100 MHz, 25 °C, chloroform-*d*) NMR of **7a** alkene/aryl region.

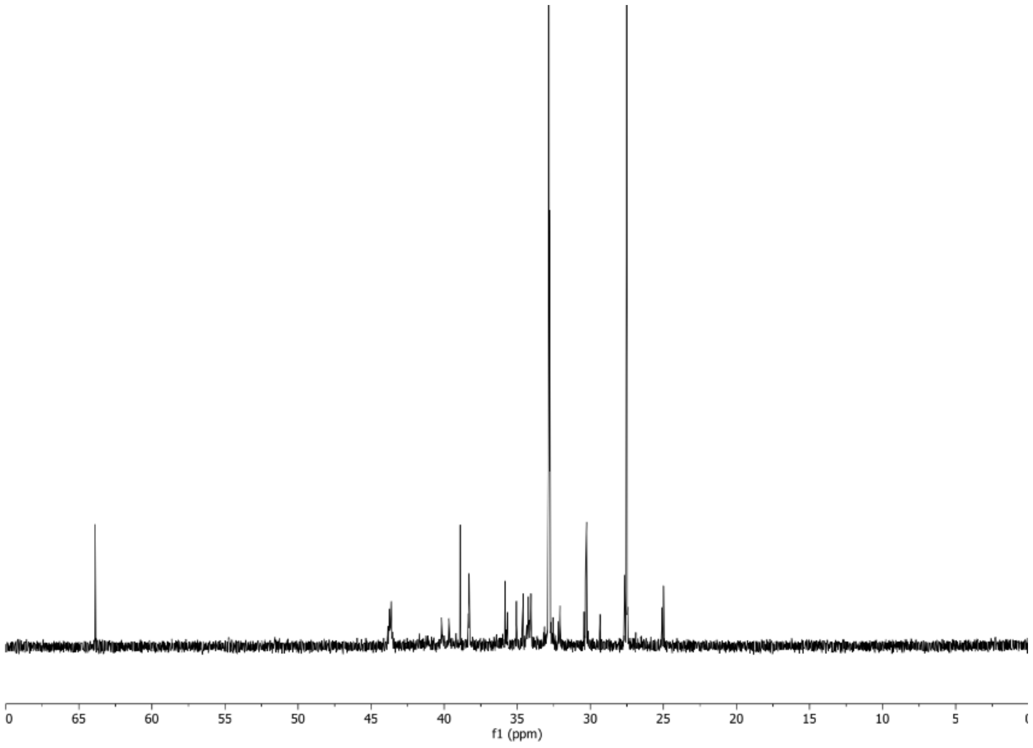

Figure S16. Representative ^{13}C (100 MHz, 25 °C, chloroform-*d*) NMR of **7a** aliphatic region.

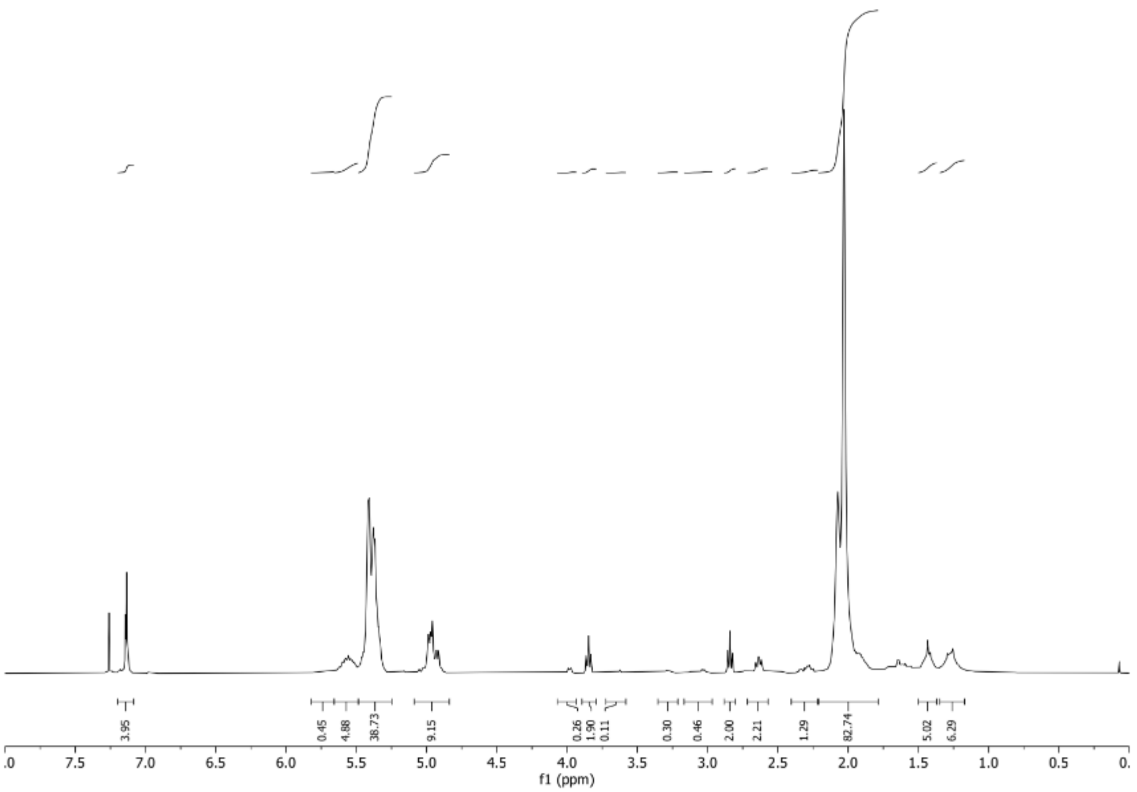

Figure S17. Representative ^{1}H (400 MHz, 25 °C, chloroform-*d*) NMR of 3-arm star polymer (**7b**).

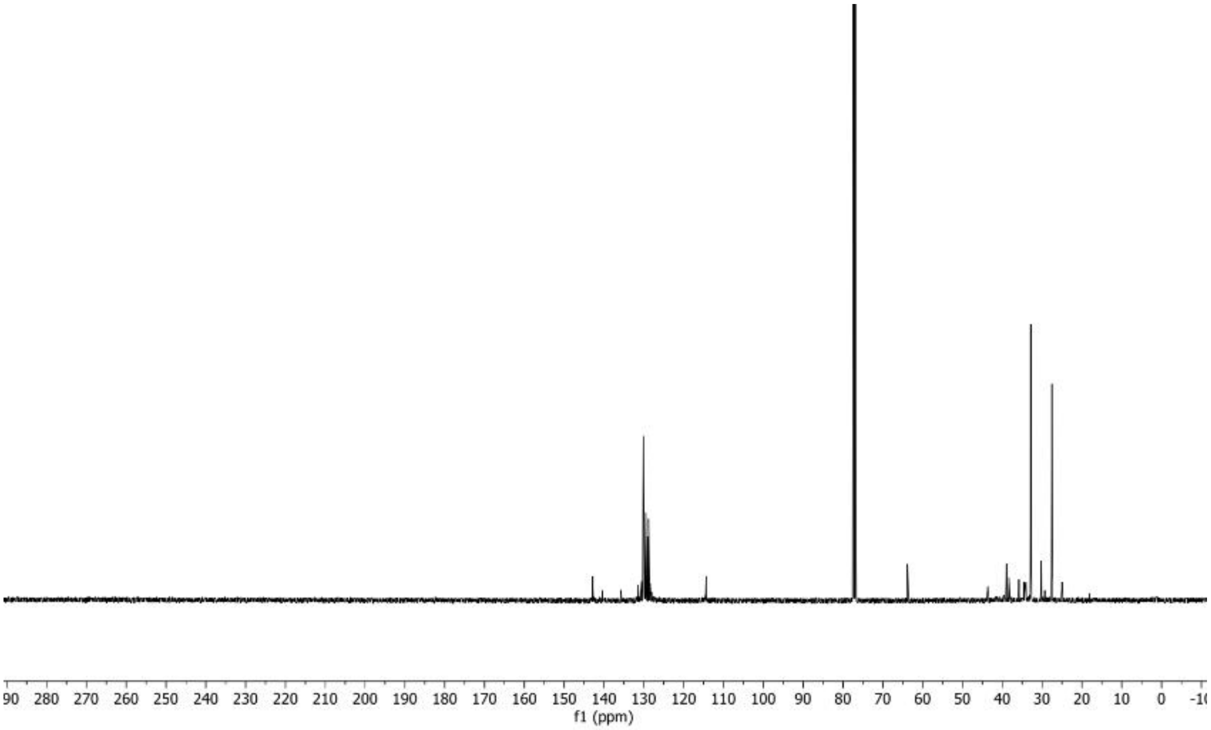

Figure S18. Representative ^{13}C (100 MHz, 25 °C, chloroform-*d*) NMR of **7b**.

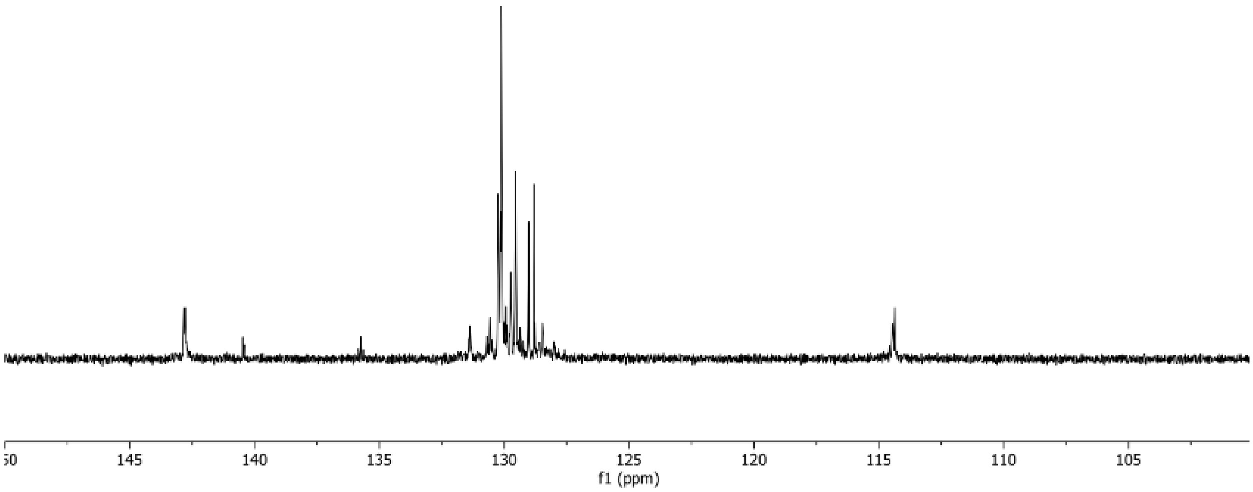

Figure S19. Representative ^{13}C (100 MHz, 25 °C, chloroform-*d*) NMR of **7b** alkene/aryl region.

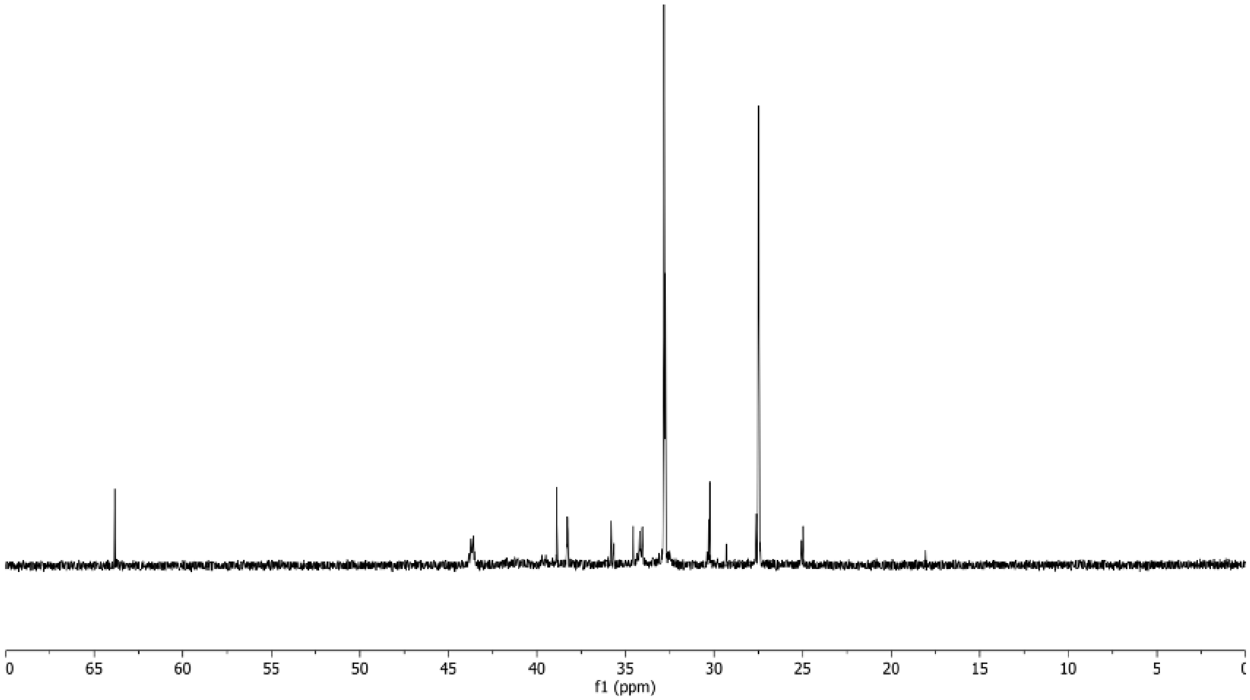

Figure S20. Representative ^{13}C (100 MHz, 25 °C, chloroform-*d*) NMR of **7b** aliphatic region.

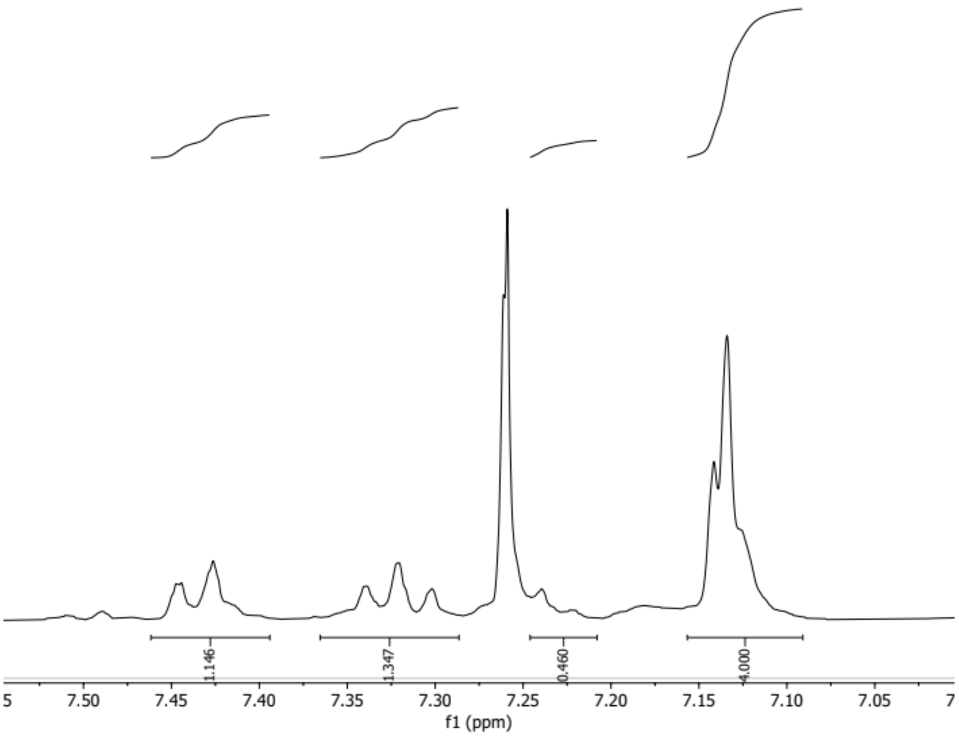

Figure S21. ^{1}H (400 MHz, 25 °C, chloroform-*d*) NMR of the aryl region of heavily DCP-contaminated **7a** (unadulterated spectrum shown in Figure 1B).

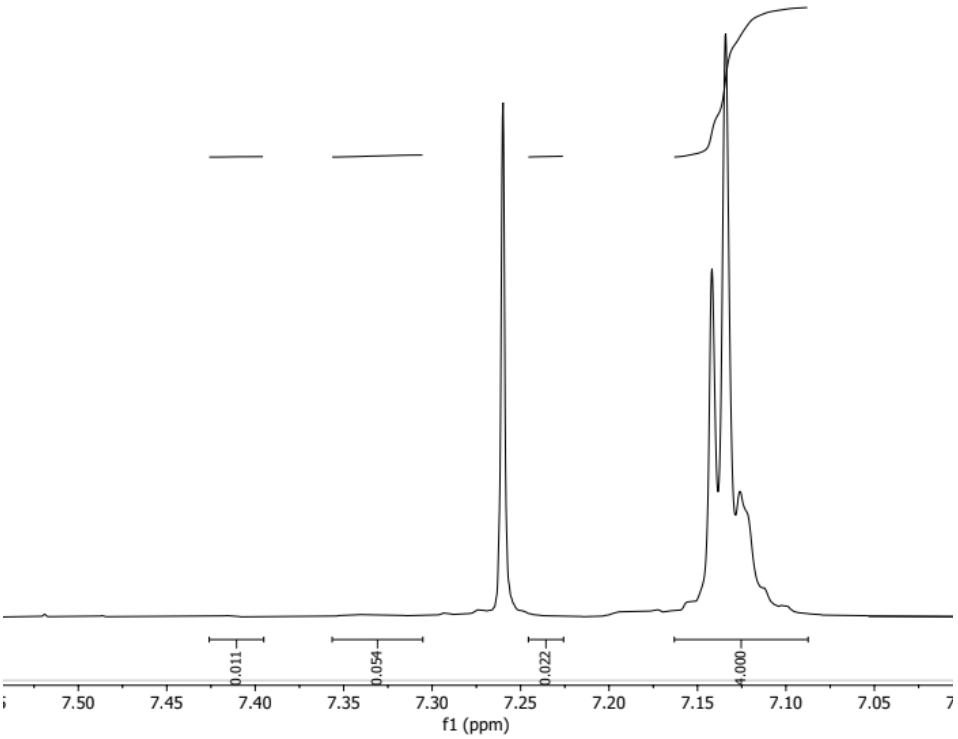

Figure S22. ^{1}H (400 MHz, 25 °C, chloroform-*d*) NMR of the aryl region of **7a** with little DCP contamination (unadulterated spectrum shown in Figure 1C).

IR Spectra of Polymers

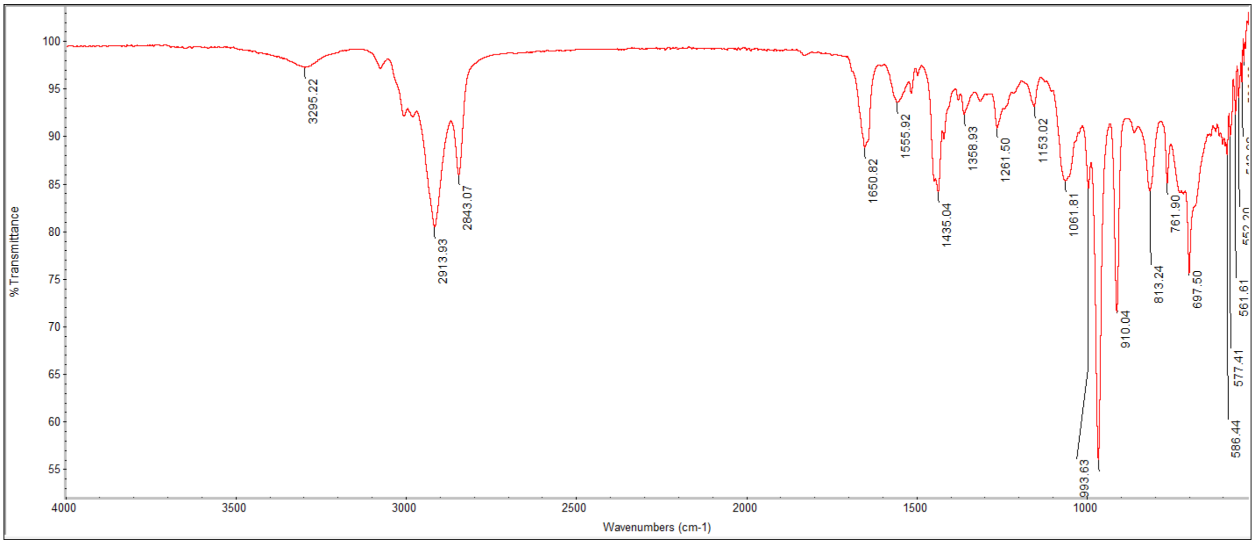

Figure S23. IR(ATR) spectrum of **7a**.

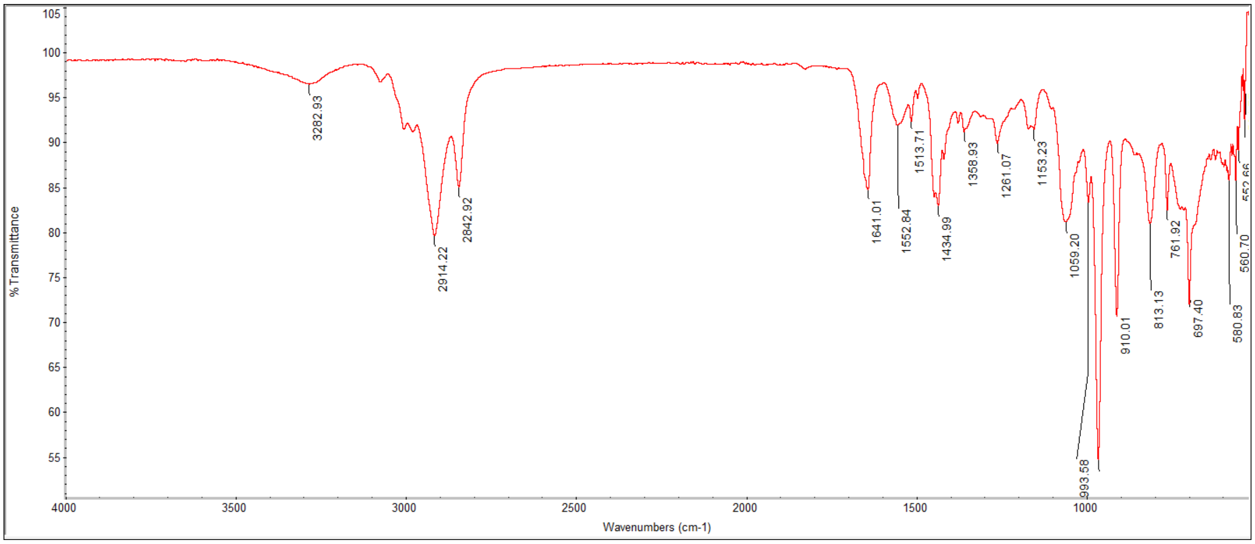

Figure S24. IR(ATR) spectrum of **7b**.

Cleavage Experiments

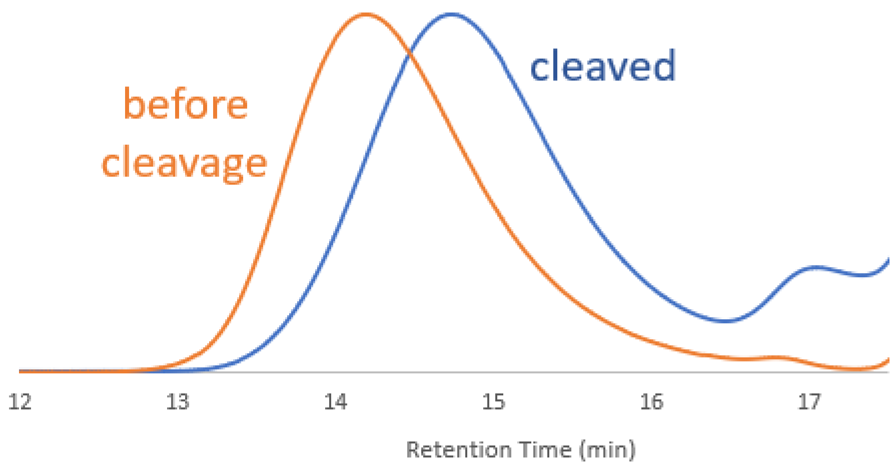

Figure S25. SEC before and after cleavage with excess AIBN in toluene at 80 °C for 4 hours of **7a**. Initial M_n = 3,298 g/mol; final M_n = 2,458 g/mol.

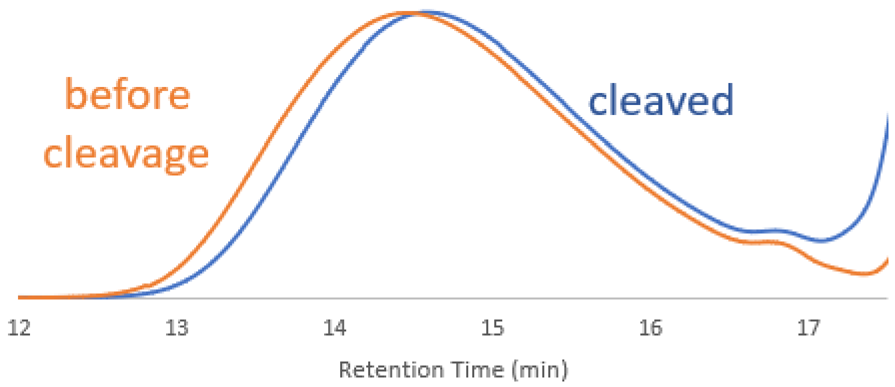

Figure S26. SEC before and after cleavage with excess AIBN in toluene at 80 °C for 4 hours of **7b**. The effect is likely less dramatic as a function of steric crowding upon the increase in arms in the core. Initial M_n = 2,499 g/mol; final M_n = 2,366 g/mol.

RAFT Experiments Using Model CTA

Scheme S1. Model RAFT of Butadiene

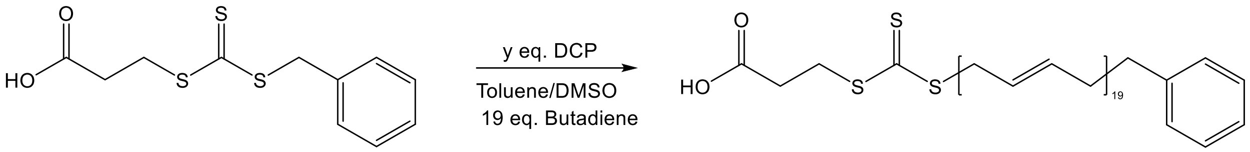

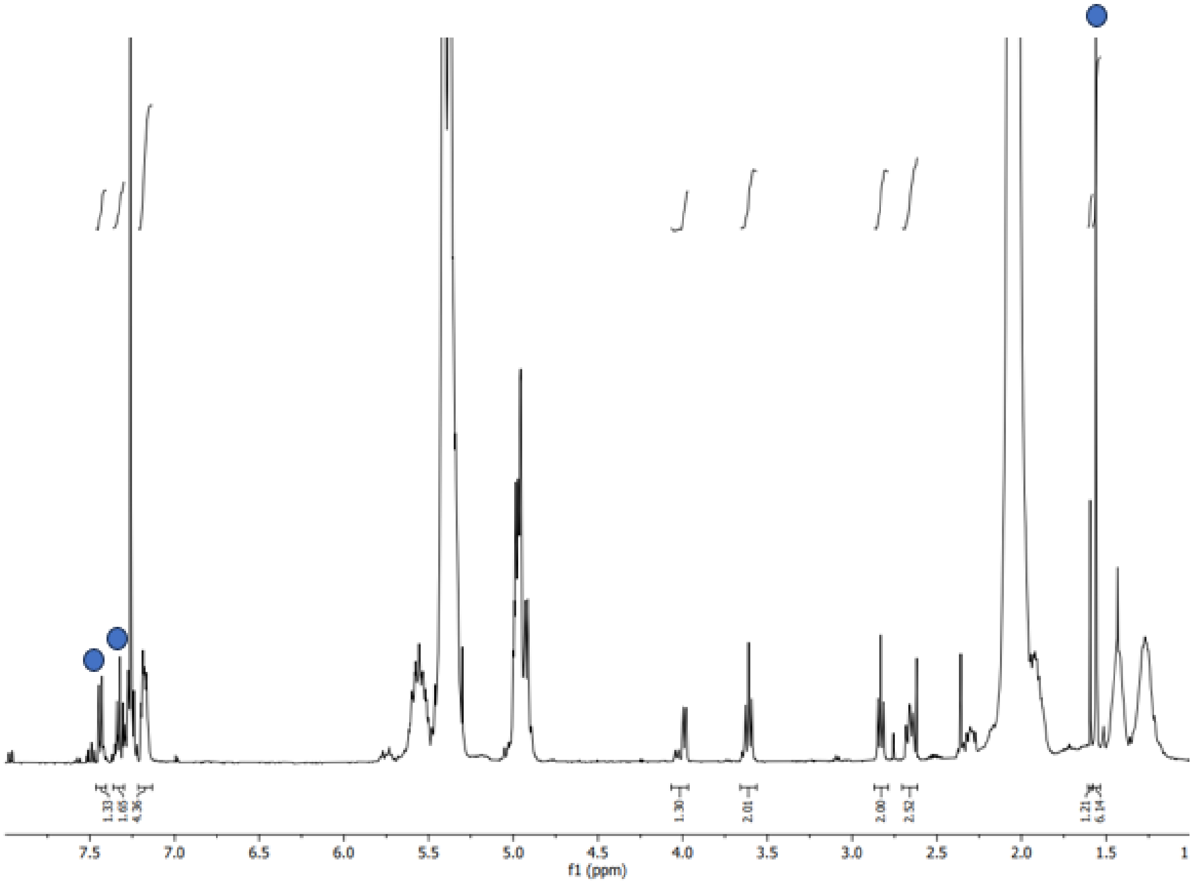

Figure S27. Representative ^{1}H (400 MHz, 25 °C, chloroform-*d*) NMR of resulting polymer with 0.50 equiv. DCP, T = 95 °C, t = 3 d. Blue circles mark DCP-derived end-groups.

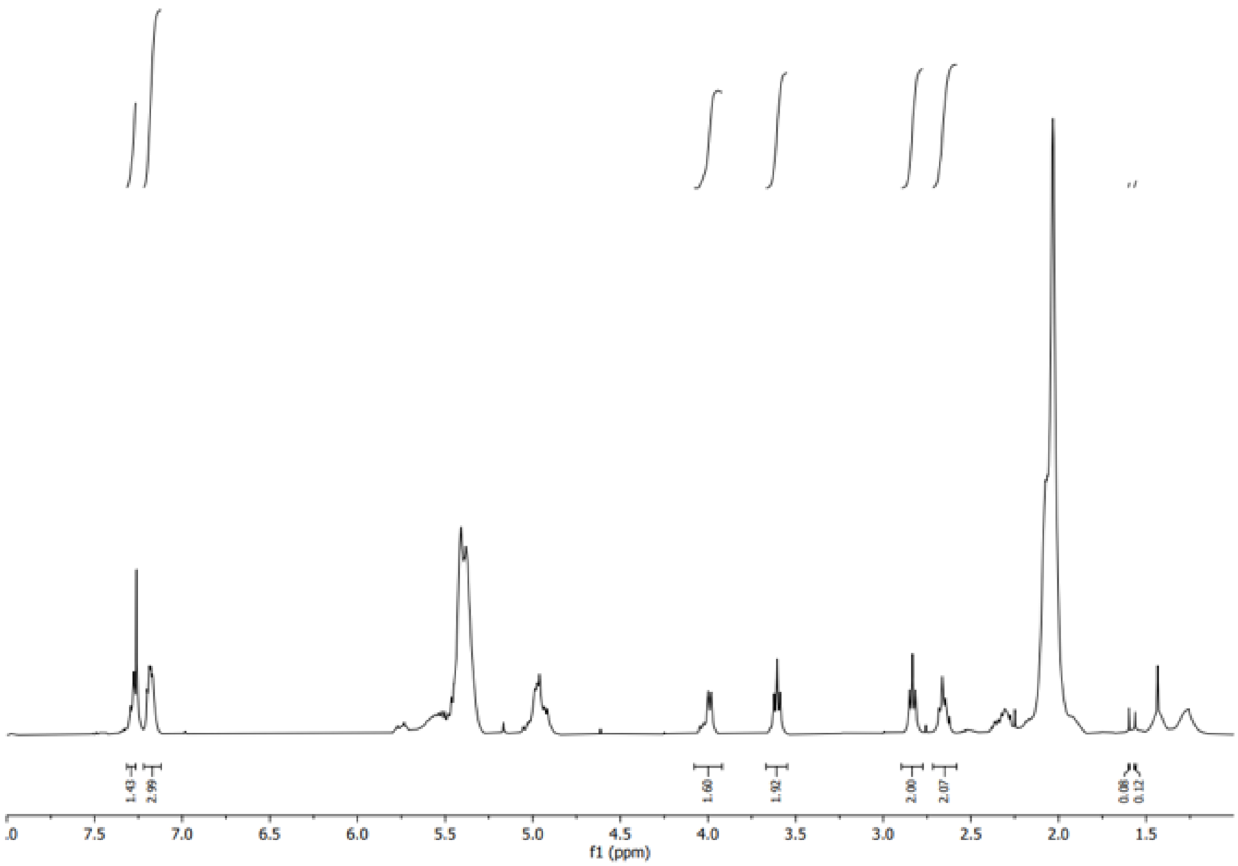

Figure S28. Representative ^{1}H (400 MHz, 25 °C, chloroform-*d*) NMR of resulting polymer with 0.05 equiv. DCP, T = 95 °C, t = 6 d.

Table S1. Summary of model polymerizations.

Expt.	Time (days)	T (°C)	Yield (g)	M_n (g/mol)[a]	Đ[a]	CTA:DCP[b]
1	3	95	0.359	1,270	1.10	1:0.06
2	6	95	0.513	1,395	1.12	1:0.02
3	9	95	0.480	1,135	1.06	1:0.02
4	6	120	0.653	1,710	1.15	1:0.01
5	3	95	1.913	6,690	1.44	1:1.02

[a]SEC with RI detection, relative to narrow MMD polystyrene standards, 25 °C, THF eluent, 1 mL/min. [b]Determined by ^{1}H NMR

Curing Experiments

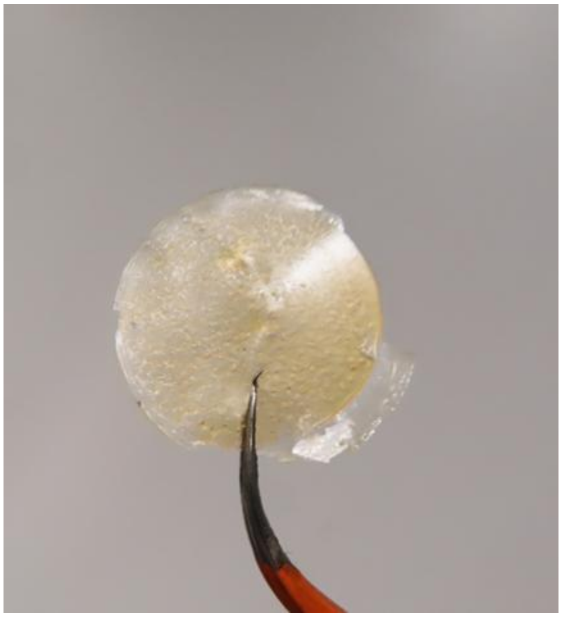

Figure S29. Cured **7b** with Desmodur N3300A.

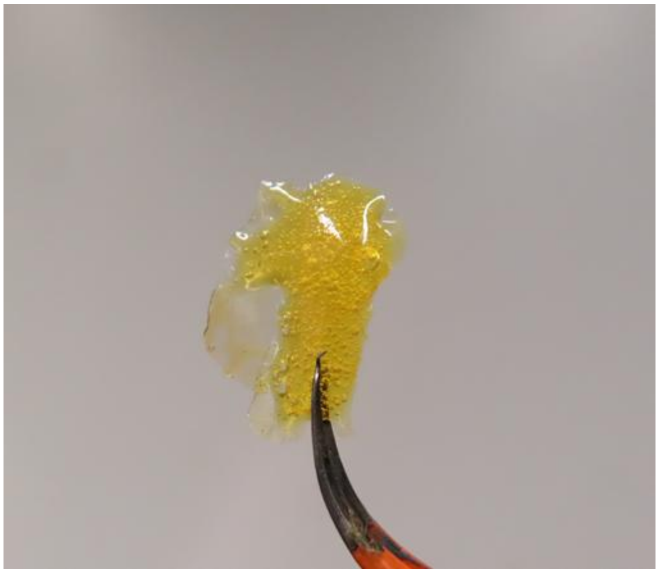

Figure S30. Cured **7b** with HDI.

DSC Data

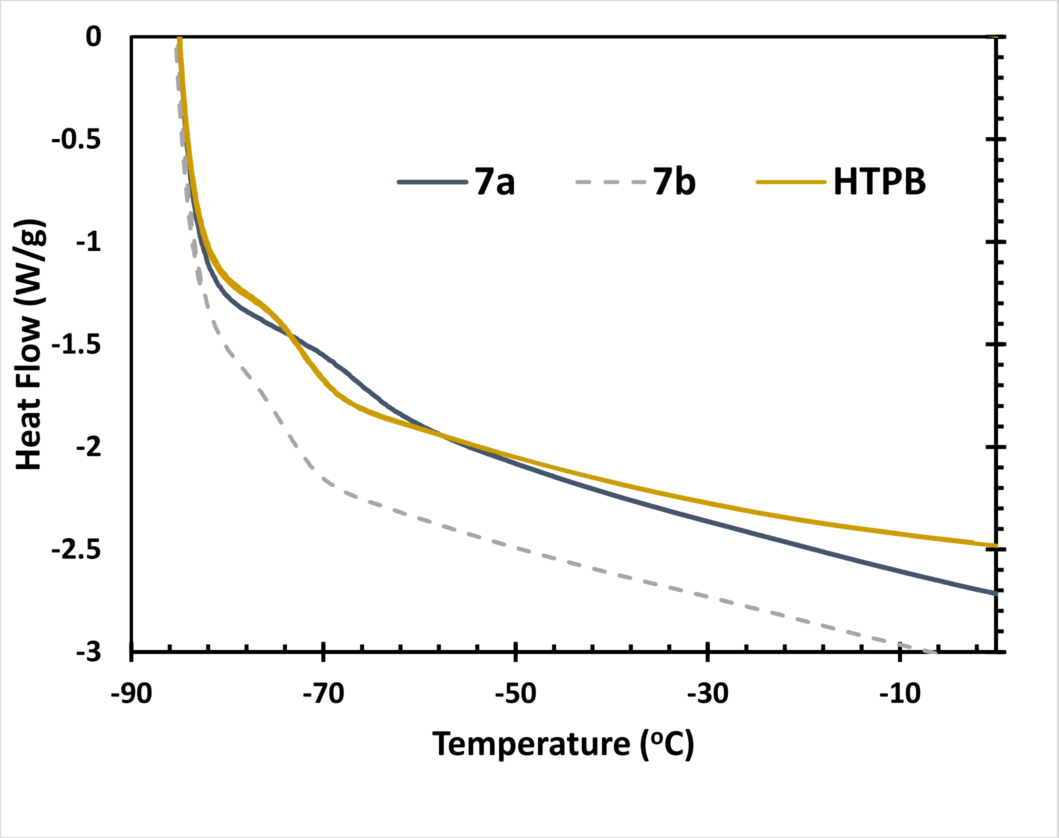

Figure S31. DSC of **7a**, **7b**, and HTPB cured with Desmodur N3300A.
